# Decreased neuronal synaptosome associated protein 29 contributes to poststroke cognitive impairment by disrupting presynaptic maintenance

**DOI:** 10.7150/thno.54210

**Published:** 2021-03-04

**Authors:** Weijie Yan, Jiahui Fan, Xia Zhang, Huimeng Song, Rongqi Wan, Wei Wang, Yanling Yin

**Affiliations:** Department of Neurobiology, Department of Physiology and Pathophysiology, Key Laboratory for Neurodegenerative Disorders of the Ministry of Education, School of Basic Medical Sciences, Capital Medical University, Beijing 100069, PR China.

**Keywords:** ischemic stroke, SNAP29, synaptic vesicle release, synaptic function, cognitive impairment

## Abstract

**Background:** Poststroke cognitive impairments are common in stroke survivors, and pose a high risk of incident dementia. However, the cause of these cognitive impairments is obscure and required an investigation.

**Methods:** Oxygen-glucose deprivation (OGD) model and middle cerebral artery occlusion (MCAO) model were used to imitate *in vitro* or *in vivo* acute cerebral ischemia, respectively. The differentially expressed synaptosome associated protein 29 (SNAP29)-interacting proteins upon ischemia and reperfusion were analyzed with bioinformatics analysis and the results indicated that the changes of SNAP29 after acute ischemia were mainly involved in the synaptic functions. The outcomes of SNAP29 reduction were assessed with SNAP29 knockdown, which mimicked the distribution of SNAP29 along neuronal processes after acute ischemia. Using the whole-cell patch clamp recording method and transmission electron microscope, the pre-synaptic function and readily releasable pool (RRP) were observed after SNAP29 knock down. Using photogenetic manipulations and behavioral tests, the neuronal projection and cognitive functions of mice with SNAP29 knock down in hippocampus CA1 region were evaluated.

**Results:** It was found that SNAP29 protein levels decreased in both *in vitro* and *in vivo* ischemic models. Further, the SNAP29 reduction wasn't associated with impaired autophagy flux and neuronal survival. When SNAP29 was knocked down in primary cortical neurons, the frequency of AMPARs-mediated mEPSCs, but not the amplitude, significantly decreased. Meanwhile, the mice with SNAP29 knockdown at CA1 region of hippocampus developed an impairment in hippocampus-mPFC (middle prefrontal cortex) circuit and behavioral dysfunctions. Moreover, the size of RRP at presynaptic sites was diminished.

**Conclusion:** Since SNAP29 protein levels didn't significantly influence the neuronal survival and its decrease was sufficient to disturb the neural circuit via a presynaptic manner, the SNAP29-associated strategies may be an efficient target against poststroke synaptic dysfunction and cognitive deficits.

## Introduction

Stroke is one of the main causes of death and disability worldwide. More than 50% of stroke survivors face distinct and persistent cognitive deficits 1, 2, along with increased disability and depression in the long term 3, 4. Up to 25% of stroke survivors with cognitive impairment have confirmed dementia within 3 years after stroke 5. Furthermore, 10% of stroke survivors develop dementia after the first stroke, and over 33% develop dementia after a recurrent stroke 6. Thus, an efficient target for intervention in short- and long-term poststroke cognitive impairment is urgently required.

To date, the search for the mechanisms responsible for the onset of poststroke dementia has not been productive. Early biomarkers for poststroke cognitive impairment have been identified, such as increased levels of soluble receptor for advanced glycation end products (sRAGE) and β-site APP cleaving enzyme 1 (BACE1) 7. sRAGE contributes to protection against stroke via its influence on oxidative stress 8. Zhang et al. reported that amyloid-β expression increases after experimental ischemia of the brain 9, which can be regulated by BACE1 activity 10. Additionally, the presence of an epsilon4 allele in apolipoprotein E (APOE), which influences lifetime cholesterol levels, is related to greater progression in cognitive decline after stroke 11, 12. Downregulated autophagy may also decrease the risk of poststroke dementia by suppressing ischemia-induced neuroamyloidogenesis 13. In addition, inflammatory reactions have been regarded as possible risk factors for poststroke dementia 14, 15. Interleukin-17 regulatory pathways may provide intervention targets to improve the recovery of poststroke long-term cognitive impairment 16. However, outside the thrombolytic therapeutic window, there is no effective treatment to ameliorate long-term cognitive dysfunction after stroke.

SNAP29, a member of the SNAP25 family, was initially regarded as a ubiquitously expressed syntaxin-binding protein. A growing body of evidence suggests that SNAP29 modulates membrane fusion at multiple cellular localizations in the process of intracellular trafficking, such as endocytosis and recycling 17. It was reported that SNAP29 accelerates endosomal trafficking of insulin growth factor 1 receptor (IGF-1R) and downregulates active receptors in CHO cells 18. In HeLa cells, alternative EHD1 and syndapin II cooperation with SNAP29 was shown to influence internalization of transferrin receptors (TFR) 19. Additionally, SNAP29 plays a vital role in the fusion of autophagosomes and lysosomes 20. Syntaxin 17 (STX17), SNAP29, and the lysosomal R-SNARE VAMP8 are all required for the fusion of autophagosomes and lysosomes in HeLa cells 21, 22. In this process, SNAP29 is possibly derived from the cytoplasm combined with STX17 and is crucial for priming fusion with VAMP8 19, 23. In addition, SNAP29 facilitates specialized secretion. SNAP29 deficiency impairs Golgi trafficking and secretion. SNAP29 has also been directly associated with unconventional secretion of interleukin-1β (IL-1β) via an autophagy-associated manner upon lysosomal damage 24-26. Further, SNAP29 may modulate synaptic vesicle fusion by regulating SNARE complex disassembly or replacing SNAP25. Thus, SNAP29 disruption may potentially interfere with synaptic vesicle transmission, which is vital for ongoing synaptic transmission 27. Alteration of SNAP29 has been found to be important in neurological disorders. *Snap29* gene has been proposed as a candidate driver of 22q11.2 synaptic pathology related to schizophrenia (SCZ) and autism spectrum disorder (ASD) 28. Indeed, loss of function of SNAP29 in the fetal process is regarded as one of the main reasons for the synaptic pathology of SCZ and ASD 29, 30. As synaptic dysfunction arises before neuronal loss in many neurodegenerative diseases, this suggests a strong pathological correlation with cognitive impairment 31, 32. However, whether SNAP29-mediated synaptic transmission is vital for cognitive functions remains to be revealed.

Studies have shown that autophagic flux is impaired in the late stage of ischemic stroke 33, 34. As SNAP29 is a member of the SNARE complex mediating fusion of autophagosomes and lysosomes, in this study we investigated the protein levels of the SNARE complex and found sustained reduction in SNAP29 after stroke. Ischemic exposure from OGD or MCAO insults reduced presynaptic levels of SNAP29 protein. We used optogenetic methods to evaluate whether there was dysfunction in the neural circuitry after SNAP29 reduction. We found that SNAP29 reduction induced cognitive dysfunction. Therefore, disruption of the function of SNAP29 was sufficient to cause cognitive impairment in mice after cerebral ischemia. To our knowledge, there is no report on the changes to SNAP29 after stroke and its associated significance, so our study suggests a novel therapeutic target against cognitive impairment following cerebral ischemia via maintaining the function of SNAP29.

## Materials and Methods

### Antibodies and Reagents

All the used antibodies in this study are listed in the **Table 1**, and reagents are listed in the **Table 2**.

### Primary cortical neuron culture

The primary cultured cortex neurons were obtained from new-born Wistar rats (within 24 h, Beijing Vital River Laboratory Animal Technology, Beijing) regardless of gender. The cerebral cortex of Wistar rats was placed into cold Hank's balanced salt solution. Tissues were trypsinized at 37 °C for 15 min. Planting medium (DMEM with 10% horse serum and 10% fetal bovine serum) was used to stop action of trypsin. Tissues were disassociated by repeated pipetting, and the supernatant was filtered. Cells were planted in culture dishes, and planting medium was replaced with culture medium (Neurobasal 48.5 mL with B27 1 mL and GlutaMax 0.5 mL) 4 h later. Primary neurons were cultured in 5% CO_2_ at 37 °C with media changed every 3 days. Cytarabine (10 μM) was added into the medium on the third day to suppress the proliferation of glial cells. The neurons were used into experiments on day 12. In this study, the usage of animals was approved by the Institutional Animal Care and Use Committee (IACUC). The ethical code is AEEI-2018-024.

### OGD treatment of cortical neurons

OGD medium (DMEM, no glucose) was preheated at 37 °C. The rat neuron culture medium was substituted with OGD medium, and culture dish was put into an incubator with 2% oxygen (5% CO_2_, 2% O_2_ and 93% N_2_). After OGD treatment, the OGD medium was replaced with culture medium, and the neurons were returned to the standard culture environment (i.e., reperfusion conditions). Unless otherwise mentioned, OGD treatment was 1 h in duration, and the duration of reperfusion varied; the treatment conditions are referred to using the format “OGD/R 12 h”, which indicates 1 h of OGD followed by 12 h of reperfusion.

For the groups with MG132 treatment, MG132 (10 μM) was added into medium 6 h before OGD exposure 35. In the following OGD and reperfusion, MG132 was also added into OGD medium and culture medium.

### MCAO model

Mice used in this experiment were C57BL/6 mice (Beijing Vital River Laboratory Animal Technology, Beijing), which were male, 6-8 weeks old and 20-22 g in weight. To avoid the disturbance of female sex steroids to ischemic injuries, male mice were selected for MCAO experiment 36, 37. The mice were reared at 22 ± 3 °C, 50-55% humidity and 12 h circadian rhythm. This experiment involving animals were approved by the Institutional Animal Care and Use Committee (IACUC). The ethical code is AEEI-2017-099. Mice were anesthetized with 20% urethane. A midline cervical incision was made. The right external carotid was freed and right external carotid artery was tied. A silicone thread embolism was inserted into the cut external carotid artery and pushed into the origin of middle cerebral artery to occlude it. After 1 h of MCAO, the silicone thread embolism was removed, and the blood flow was restored 38.

### Transfection of cortical neurons with lentiviruses

pLenti-CMV-*Snap29*, pLKD-U6-shRNA, pLenti-Ubc-EGFP-Map1lc3b, and pLenti-CMV-mCherry-GFP-LC3B were provided by OBiO Technology Corporation (Shang Hai, China). Viruses (MOI=10) were added to the culture medium of cortical neurons on culture day 6. After 24 h, the medium with lentiviruses was replaced with fresh medium. The neurons were used for experiments until the 6 days after virus infection.

### Adeno-associated virus (AAV) injection

pAAV-CMV-hChR2(H123R)-mCherry-U6-shRNA (*Snap29* knockdown) was obtained from OBiO Technology Corporation (Shang Hai, China), and pAAV-CMV-RFP-U6-shRNA (*Snap29*) was provided by Vigene Bioscience (Shan Dong, China). Before injection, the mouse was anesthetized with 20% urethane and fixed on a stereotaxic apparatus. The scalp was cut along the midline, and the bregma was marked. A craniotomy was performed with a bone drill, and the virus was injected into the hippocampus CA1 area (relative to bregma: -2.7 mm anteroposterior, ± 3.2 mm mediolateral, and 2.7 mm dorsoventral). The virus was injected using a 2 μL microsyringe at a rate of 0.2 μL/min 39. The sequences of the shRNAs and their targets on the sequence of interest are presented in **Table 5**.

### Lactate dehydrogenase (LDH) release assay

After OGD and OGD/R 1 h, culture medium, OGD medium, and reperfusion medium were collected, respectively, and stored on ice. Assay buffer was added to lyse the cells. The cells were homogenized by ultrasound at 4 °C, and then the lysate was centrifuged at 10000 × *g* for 15 min at 4 °C. The supernatant was collected in a new tube (n = 3). Then, an LDH assay kit was used to assess LDH content in the medium 40. The experiment was repeated three times.

### Cell viability assay

Primary cortex neurons were cultured in 96-wells plate (5 × 10^4^ cells/well). After OGD, OGD/R 1 h or OGD/R 12 h, cell viability was assessed with a Cell Counting Kit (CCK) assay (n = 9). The absorbance at 450 nm was measured using a microplate reader.

### Real-time quantitative PCR (RT-qPCR)

Total RNA was isolated from primary cultured neurons with TRIzol reagent. A NanoDrop (Thermo Scientific, USA) was used to measure the concentration of total RNA and check its purity. cDNAs were prepared with Revert Aid First Strand cDNA Synthesis Kit. mRNA levels were measured by RT-qPCR using the specific primers listed in **Table 3** and SYBR green. The value of 2^-ΔΔCt^ was calculated and the results are presented as fold change relative to β-actin. The experiment was repeated three times.

### Transmission electron microscopy (TEM)

Neurons were fixed with 2.5% glutaraldehyde at 4 °C and then postfixed with 2% osmium tetroxide. The cells were dehydrated with cold graded ethanol and then rinsed in propylene oxide. Next, the neurons were embedded with Epon 812 medium. Sections (90 nm) were cut and stained with 0.2% lead citrate and 1% uranyl acetate. Images were acquired with an electron microscope (Tecnai Spirit 120 kV, FEI, USA) at 80 kV.

SNAP29-knockdown mice were perfused with 4% polyformaldehyde, and then brain tissue was postfixed with 4% polyformaldehyde for 2 h. The brain was cut into 100 μm slices with a vibrating microtome, and the hippocampus was separated out. The tissues were dehydrated with cold graded ethanol and then rinsed in propylene oxide. The tissues were embedded with LX-112 medium. 90 nm sections were cut and stained with 0.2% lead citrate and 1% uranyl acetate. Images were captured with an electron microscope (HT7700, Hitachi, Japan) at 80 kV. These experiments were repeated three times.

### Immunofluorescence staining

After treatment, cells were fixed with 4% polyformaldehyde for 30 min, and then 0.3% Triton X-100 was added for 15 min. The cells were washed with 1 × PBST (10 mM Na_2_HPO_4_, 1.8 mM KH_2_PO_4_, 2.7 mM KCl, 137 mM NaCl, pH 7.4, 0.1% Tween-20) and blocked with 5% BSA for 1 h. The cells were incubated with primary antibody for 24 h at 4°C and then secondary antibody for 2 h at room temperature. Mounting medium containing DAPI was added. Immunofluorescence images were acquired with a laser scanning confocal microscope (TCS SP8 STED, Leica) and a structured-illumination microscope (A1 N-SIM STORM, Nikon, Japan). Fluorescence intensity and co-localization analyses were conducted with Imaris 9.3.1 (Oxford Instruments Group, UK). The experiment was repeated three times and the number of selected neurons was not less than 6.

### Western blotting

For cultured cells, 30 μL RIPA buffer containing protease inhibitor and phosphatase inhibitor (Roche, Switerland) was added to each well of a 6-well plate and the cells were scraped. For tissue samples, 100 mg cerebral cortex was thawed on ice in 1 mL RIPA buffer. Brain tissues were collected from areas near the infarct region in the MCAO model, which was verified by 2,3,5-Triphenyl-tetrazolium chloride staining. The cells/tissues were homogenized by ultrasound at 4 °C, and then the lysate was centrifuged at 13,800 × *g* for 10 min at 4 °C. The supernatant was transferred into a new tube. A BCA Protein Assay Kit was used to quantify the total protein. Proteins (30 μg) from each sample were separated by sodium dodecyl sulfate polyacrylamide gel electrophoresis (SDS-PAGE) and transferred onto polyvinylidene difluoride membranes. The membranes were blocked with 10% no-fat milk for 1 h and then incubated with antibodies. After incubation with primary and secondary antibodies, a chemiluminescence kit was employed to detect the signals. The experiments were repeated no fewer than four times.

### Co-immunoprecipitation (co-IP), mass spectrometry, and bioinformatics analysis

Co-IP was performed with Pierce Classic IP Kit (Thermo Scientific, USA) following the manufacturer's instructions. Primary cultured neurons were lysed with 200 μL lysis buffer for 10 min. The lysate was transferred to a new micro-centrifuge tube and centrifuged at 13,000 × g for 10 min at 4 °C. Protein concentration was measured with BCA Protein Assay Kit (Thermo Scientific, USA). Control IgG or anti-SNAP29 antibody were added to the supernatant, and the samples were incubated overnight at 4 °C on a rotating device (DR-Mix, HERO, Italy). Then Protein A/G Plus Agarose was added to the lysate/antibody sample and incubated at room temperature for 1 h. The beads were washed 3 times with lysis buffer, resuspended with 50 μL 2 × Non-reducing Lane Marker Sample Buffer, and then boiled at 100 °C for 10 min. The samples were cooled to room temperature, and the protein (20 μL) were separated by the SDS-PAGE with SDS-PAGE running buffer (Applygen Technologies, Beijing, China). When all samples entered the separation gel, electrophoresis was stopped. Protein bands were cut from the gel and prepared for liquid chromatography with tandem mass spectrometry (LC-MS/MS).

LC-MS/MS analysis was carried out by a central laboratory of Capital Medical University. For separation, the samples were loaded on an Acclaim PepMap RSLC C18 analytical column (75 μm × 25 cm, 2 μm particle size; Thermo Scientific, USA) following an Acclaim C18 PepMap100 nano-Trap precolumn (75 μm × 2 cm, 2 μm particle size; Thermo Scientific, USA). The flow rate was set to 300 nL/min with a linear gradient of 3-30% solvent B buffer (0.1% formic acid) over 43 min followed by 30% solvent B buffer for another 1 min. Mass spectrometry analysis was accomplished in full scan mode (350-1,600 m/z) using a QExactive Orbitrap mass spectrometer (Thermo Scientific, USA) with a mass resolution of 70,000 at 400 m/z. The 20 most intense MS2 fragments from each duty cycle were chosen for MS/MS analysis and assessed at a mass resolution of 35,000 at 400 m/z. All tandem mass spectra were performed using the higher-energy collision dissociation method. Dynamic exclusion was set to 18 s. The raw date was imported into MaxQuant software for database searching. A fold change cutoff of |log2 ratio| ≥ 0.5 was used for assess differentially expressed proteins. OmicsBean (http://www.omicsbean.cn/) was used for further analyses. GO and KEGG databases were searched for functional analysis, and STRING database was used to analyze protein-protein interactions.

### Whole-cell patch clamp recording

AMPAR-mediated miniature excitatory post-synaptic currents (mEPSCs) of cortical neurons were recorded using a whole-cell patch clamp recording method at room temperature. Patch electrodes of thick-walled borosilicate glass were pulled on a PP-83 micropipette puller (Narishige, Japan). The patch-pipette solution contained: 140 mM KCI, 10 mM HEPES, 10 mM EGTA, 2 mM MgCI_2_, 2 mM Na_2_ATP, 1 mM CaCI_2_ with a pH of 7.3 adjusted using KOH. The extracellular solution contained: 140 mM NaCI, 5 mM KCI, 1 mM MgCI_2_, 0.5 mM CaCI_2_, 10 mM glucose, 10 mM HEPES with a pH of 7.4 adjusted using NaOH. The resistance of the glass electrodes was 3-5 MΩ. To record AMPAR-mediated mEPSCs, the GABA_A_ receptor antagonist bicuculline (50 μM), the NMDA receptor antagonist 2-amino-5-phosphonopentanoic acid (50 μM) and tetrodotoxin (1 μM) were added to the extracellular solution. mEPSCs were gained at a holding potential of -70 mV for at least 5 min without synaptic stimulation (n = 11). Pyramidal neurons were selected for recording.

### Quantitative image analysis

To quantify SNAP29 reduction following shRNA transfection and OGD/R 1 h treatment, the expression levels of SNAP29 on presynaptic sites were analyzed as previously reported 41. For each image, neurons were segmented manually from background using Imaris 9.3.1, a binary map was generated, and the locations of neuronal processes were labeled. Further segmentation of the presynaptic sites was performed using custom codes (MATLAB 2019a, MathWorks, USA). The fluorescence (red channel, 594 nm) images of synaptophysin were used to detect presynaptic sites along the neuronal processes. In order to distinguish the presynaptic sites of each neuron from background and noise, three key parameters were selected: mean intensity, area and distance from neuronal processes. Before detection, all images were normalized to pixel values between 0 and 255. The normalized grayscale images were binarized using a global threshold, with nonzero pixels corresponding to presynaptic sites (foreground) and zero-value pixels corresponding to background. For each two adjoining pixels in the foreground, if they were connected along the horizontal, vertical, or diagonal direction, they were considered to be part of the same object. Since we had labeled the neuronal processes manually, the distance between each presynaptic site and the corresponding process could be calculated, and sites within 1 μm from a process were considered to belong to the neuron. Moreover, to reduce the background noise, areas of connected components smaller than 0.35 μm^2^ or larger than 10 μm^2^ were removed. After detection and segmentation of the presynaptic sites, the fluorescence intensity of SNAP29 was calculated using green channel images. Mean intensity values of SNAP29 signals from presynaptic sites were analyzed, to reflect the expression levels of SNAP29 on presynaptic sites, and their density distribution and cumulative frequency distributions were analyzed to assess the properties of SNAP29 expression under various conditions. Differences between groups were statistically analyzed with Kolmogorov-Smirnov test.

### Optogenetic stimulation and local field potential recording

Local field potential was recorded three weeks after virus (pAAV-CMV-hChR2(H123R)-mCherry-U6-shRNA (*Snap29*)) injection. 2% isoflurane was used for anesthesia induction, and 0.8% isoflurane was used for anesthesia maintenance. After anesthesia induction, the mouse was fixed on a stereotaxic apparatus and its scalp was cut along the midline. The bregma and the target location were marked. A single channel recording electrode was placed in the mPFC (relative to bregma: 1.93 mm anteroposterior, 0.5 mm mediolateral, and 1.83 mm dorsoventral) and an optical fiber was inserted into the hippocampus (relative to bregma: -2.7 mm anteroposterior, 3.2 mm mediolateral, and 2.7 mm dorsoventral). The optical fiber was connected to a bushing, which was fixed to the skull with dental acrylic. Light intensity and wavelength were calibrated before the experiment. Optical stimulation was provided by a DSSPL DRIVER (SLOC LASERS, China). After a 20 min adaptation period, local field potential was recorded with an OmniPlex® Neural Recording Data Acquisition System (PLEXON, USA). The experiment was repeated three times and the sample number was not less than 7.

### Open field test

An open field test was used to evaluate spontaneous activities, anxiety-like behavior, and emotional changes in mice. The open field chamber (50 cm × 50 cm × 30 cm) was black. Before the test, the subject mice were placed in the behavioral testing room for 30 min to acclimate to the environment. The mice were placed in the center of the chamber and allowed to move freely within the chamber. The process was recorded for 30 min 42. The total travel distance, number of entries to the central area (30 cm × 30 cm) and residence time in the central area were then recorded. After each test, 75% ethyl alcohol was used to clean the chamber. The sample number was 8 per group.

### Novel object recognition test

The test consisted of three phases: 'habituation', 'object familiarization', and 'object recognition'. Before testing, the experimental mice were placed in the behavioral testing room for 30 min to acclimate to the environment. In the 'habituation' phase, mice were placed in a chamber for 5 min to adapt to the experimental environment. Then, in the 'object familiarization' phase, two identical spherical objects were placed in the chamber and the mice were allowed to explore for 5 min. Before the 'object recognition' phase, there was a 1 h intertrial interval. Then, one of the original spherical objects was replaced with a conical object. The mice were placed in the chamber for 5 min, and the time spent sniffing each object was recorded. The sample number was not less than 7. The discriminant index was calculated as (conical object sniffing time)/(conical object sniffing time + spherical object sniffing time) × 100% 43.

### Three-chamber sociability task

A three-chamber box was used to assess general sociability and reactions to social novelty. The test consisted of three phases: 'habituation', 'sociability', and 'social novelty'. There were openings between the chambers of the box, which allowed the mice to freely explore all three chambers. Glass panels were used to close the openings during phase changes. Before testing, the subject mice were placed in the behavioral testing room for 30 min to acclimate to the environment. Each phase lasted 5 min. The experimental mouse was placed in the three-chamber box to adapt to the testing apparatus. Next, in the 'sociability' phase, an unfamiliar mouse (stranger #1) was placed in the right chamber and the time that the experimental mouse spent sniffing stranger #1 was recorded. The experimental mouse was then put back into the center chamber, and the openings were covered. Then, in the 'social novelty' phase, a second unfamiliar mouse (stranger #2) was placed in the left chamber. The glass panels were removed so that the experimental mouse was again permitted to freely explore the chambers and interact with strangers #1 and #2. The time spent sniffing each stranger was recorded. The sample size was 8 in the wildtype (WT) and negative control (NC) groups and 7 in the SNAP29 knockdown (SNAP29 KD) group. Since the experimental mouse was familiar with stranger #1 but not #2, the time that it spent sniffing stranger #2 vs. #1 revealed the subject mouse's preference for novel social interaction 44.

### Morris water maze

Morris water maze test was conducted in a black pool with a diameter of 150 cm and depth of 60 cm. The height of the platform was 40 cm, and water (22 °C) dyed white with titanium dioxide was added to just submerge the platform (such that the platform was maintained at 1 cm below the water surface). The tank was divided into four equal quadrants and spatial clues were placed at the borders of each quadrant on the pool wall above the water. The platform was fixed in the middle of one quadrant until the end of the test.

Before testing, the experimental mice were placed in the behavioral testing room for 30 min for habituation. The subject mice were trained for 6 consecutive days (4 trials per day). The trials were started at 18:00 every day. The experimental mouse was placed in a random starting position facing the pool well. The test ended when the mouse reached the platform, and then the mouse was allowed to stand on the platform for 20 s. If the mouse did not reach the platform after 60 s, the mouse was guided onto the platform and allowed to stand there for 30 s. Swimming speed was recorded on day 1, and escape latency and swimming path were recorded by video (ANY-maze, Stoelting, USA) on days 1 to 6. On day 7, the platform was removed and the mice were tested for reference memory. The experimental mouse was placed in a novel starting position opposite the aforementioned platform, and the swimming path was recorded for 60 s. The sample size was 9 in the WT group, and 8 in the NC and SNAP29 KD groups. Time in each quadrant, number of entries into each quadrant, and number of crossings over the previous site of the platform were recorded 45.

### Statistical analysis

A *t*-test was performed to compare 2 groups; One-way ANOVA was used to compare more than 2 groups, followed by pairwise multiple comparisons with Bonferroni correction. Data are expressed as mean±SEM. *P* < 0.05 was considered significant.

## Results

### SNAP29 levels in cortical neurons sustainably decreased following OGD

**Figure 1:**
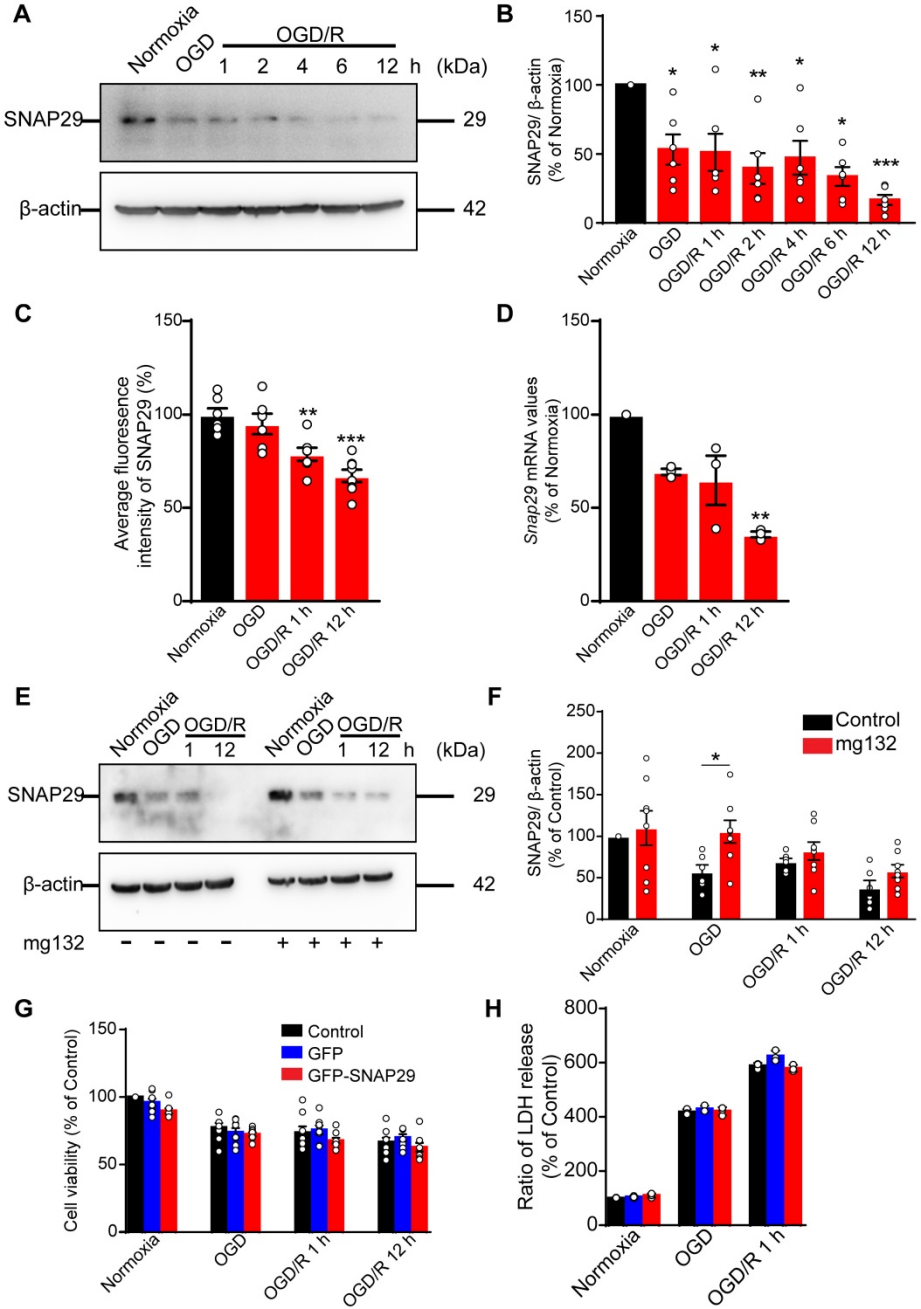
The SNAP29 protein levels significantly decreased after exposed to OGD. **A.** SNAP29 in neurons was detected following OGD exposure and during subsequent reperfusion (1, 2, 4, 6, and 12 h) with western blot assay, and quantitative analysis was shown in **B** (*p < 0.05, **p < 0.01, and ***p < 0.001 vs. normoxia group, n = 6 per group, one-way ANOVA; Bonferroni post-test).** C.** Immunofluorometric assay of SNAP29 levels following OGD/R exposure (**p < 0.01 and ***p < 0.001 vs. normoxia group, n = 6 per group, one-way ANOVA; Bonferroni post-test). **D.** RT-qPCR analysis of *Snap29* mRNA in neurons following OGD/R exposure (**p < 0.01, n = 3 per group, one-way ANOVA; Bonferroni post-test). **E.** SNAP29 in neurons was detected after pretreatment of MG132 (10 µM for 6 h) and then OGD/R (0, 1 and 12 h) exposure using western blot, and quantitative analysis was shown in **F** (*p < 0.01, n = 6 per group, Studentʼs t-test; one-tailed). **G.** After over-expressed with SNAP29 plasmid, the survival of neurons upon OGD or during subsequent reperfusion were detected with CCK8 assay (n = 9 per group, one-way ANOVA; Bonferroni post-test).** H.** The survival of SNAP29 overexpressed neurons following OGD/R exposure were detected with LDH assay (n = 3 per group, one-way ANOVA; Bonferroni post-test). Data are shown as mean and SEM.

To assess the changes in SNAP29 during OGD, we measured the SNAP29 protein levels by western blotting. SNAP29 protein levels decreased following 1 h of OGD, and the low levels were sustained through 12 h of reperfusion (**Figure 1A-B**). Similarly, the fluorescence intensities of SNAP29 in a high-content assay were significantly reduced upon OGD/R 1 h and OGD/R 12 h (**Figure 1C**).

To identify the basis of this reduction in SNAP29 protein levels, *Snap29* mRNA expression was quantified using RT-qPCR. We found that *Snap29* mRNA levels decreased in the OGD/R 1 h and OGD/R 12 h groups (**Figure 1D**), suggesting that the initial reduction in SNAP29 protein levels following OGD was caused by disrupted homeostasis due to changes in posttranslational processes, and the decreased biogenesis of SNAP29 contributed to the reduced SNAP29 protein levels in the reperfusion stage. As a main protein degradation pathway, the effect of ubiquitin-proteasome-pathway on SNAP29 reduction was evaluated by western blotting. MG132, an inhibitor of ubiquitin-proteasome-pathway, restored SNAP29 levels during OGD but did not have a significant effect on SNAP29 levels during subsequent reperfusion (**Figure 1E-F**). These results suggest that the oxygen-dependent reduction in SNAP29 levels resulted from degradation via the proteasome system in the early stage and decreased biogenesis in the reperfusion stage.

We next explored the role of SNAP29 in neuronal survival following OGD and OGD/R. First, we assessed neuronal viability using a CCK assay, and found that neuronal survival decreased following OGD, OGD/R 1 h, and OGD/R 12 h (**Figure S1**). Next, we overexpressed SNAP29 using a *Snap29* overexpression lentivirus (**Figure S2**). Under these conditions, SNAP29 protein levels did not determine neuronal fate following OGD or OGD/R, as determined by both CCK and LDH assays (**Figure 1G-H**). Based on these results, SNAP29 protein levels were maintained at a low level during OGD and OGD/R but were not significantly responsible for neuronal survival.

### SNAP29 reduction did not contribute to autophagic dysfunction

Dysfunction of autophagy machinery contributes to neuronal survival during ischemia and reperfusion. SNAP29 was reported to be a vital protein of the SNARE complex for fusion of autophagosomes and lysosomes 20. We therefore studied the effects of SNAP29 reduction on autophagy machinery. Autophagy levels were assessed by measuring microtubule-associated protein 1 light chain 3 (LC3) conversion and p62 protein levels in cortical neurons following OGD/R of various durations by western blotting. We found that the LC3II/LC3I ratio increased following OGD and OGD/R 1 h, but not in the late stage (OGD/R 2 h, OGD/R 4 h, OGD/R 6 h, and OGD/R 12 h) (**Figure S3A-B**). Meanwhile, p62 protein significantly accumulated only in the OGD/R 12 h group (**Figure S3A-C**), which indicated that autophagic flux may be impaired only after 12 h of reperfusion. To further confirm these changes in autophagic flux, cortical neurons were transfected with a Lenti-EGFP-mCherry-LC3 fusion protein. We were able to identify LC3 puncta by SIM under all four examined experimental conditions (normoxia, OGD, OGD/R 1 h, and OGD/R 12 h) (**Figure S3D**). The numbers of both EGFP^+^mCherry^+^ and EGFP^-^mCherry^+^ puncta in the neurons increased following OGD and OGD/R 1 h, but not following reperfusion of longer duration (**Figure S3E-F**). These results indicate that autophagic flux was augmented by OGD or OGD/R 1 h. Additionally, there was no significant suppression of autophagic flux (**Figure S3A-C**) during the whole OGD/R 12 h period, and SNAP29 reduction was not accompanied by changes in autophagic flux in the first step.

Next, the cortical neurons were transfected with Lenti-EGFP-LC3 and then stained for SNAP29 with anti-SNAP29 antibody (**Figure S4A**). Upon examination by SIM, more EGFP-LC3 puncta was observed in the neurons following OGD and OGD/R 1 h exposure, relative to normoxic cells (**Figure S4B**). However, the ratio of EGFP-LC3 puncta colocalizing with SNAP29 puncta to total EGFP-LC3 puncta was not significantly different among the three experimental groups (**Figure S4C**). Moreover, line tracing analysis showed that colocalization between SNAP29 spots and LC3 puncta did not change even with significant accumulation of LC3 puncta (**Figure S4D**). These results further support the idea that SNAP29 reduction was not involved in the regulation of autophagic flux following OGD or OGD/R. Interestingly, SNAP29 localization guaranteed maintenance of autophagic functions following ischemic insults in the presence of reduced SNAP29 protein levels.

### SNAP29 reduction mainly occurred at synaptic sites

**Figure 2:**
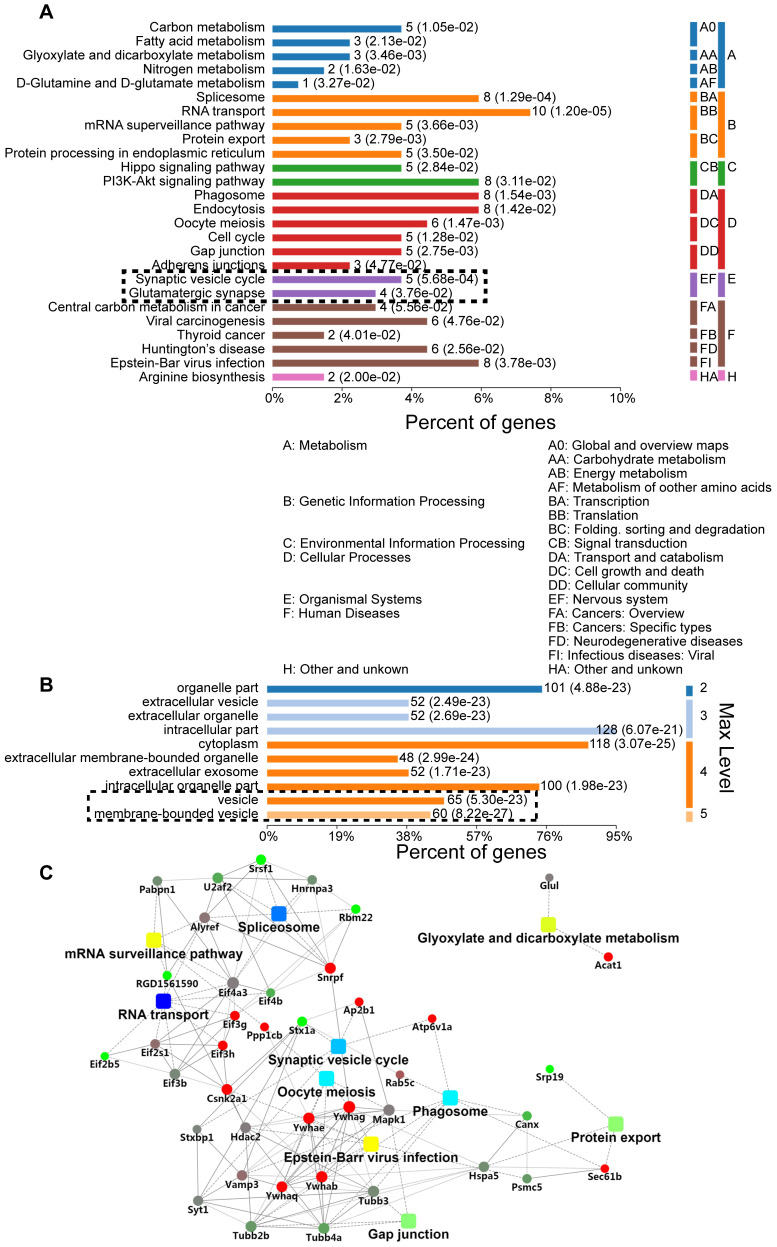
GO analysis of differentially expressed SNAP29-interacting proteins, using Database for Annotation, Visualization, and Integrated Discovery (DAVID). **A.** The most significantly enriched Kyoto Encyclopedia of KEGG pathway of the differentially expressed proteins was cytoplasm transportation. The synaptic vesicle cycle and glutamatergic transmission processes are particularly noteworthy, given the use of neurons. **B.** Differentially expressed SNAP29-interacting proteins mainly located in the cytoplasm or associated with vesicles. **C.** The identified SNAP29 interacting differentially expressed proteins in PPI network were involved in the splicesome, RNA transport, oocyte meiosis, phagosome, glyxylate and dicarboxylate metabolism, gap junction, protein export and synaptic vesicle cycle, which suggests that SNAP29-associated synaptic function may be of great importance in the OGD and/or reperfusion process.

We further explored the role of sustained SNAP29 reduction following OGD/R. Specifically, we used a co-IP assay to isolate SNAP29-interacting proteins under normoxia and OGD conditions, mass spectrometric analysis to identify the SNAP29-interacting proteins, and bioinformatics analysis to characterize differentially expressed SNAP29-interacting proteins in terms of their metabolic processes, functional categories, subcellular distributions, signaling pathways, and networks. Three hundred and thirty proteins co-precipitated with SNAP29. Among these, 129 proteins expressed in neurons according to NCBI database were manually selected for further analysis. We found that the most commonly identified metabolic processes of SNAP29-interacting proteins were related to the Gene Ontology (GO) categories of transportation, synaptic vesicle transportation and PI3K-Akt signaling (**Figure 2A**). Their binding activity showed that the differentially expressed proteins were mainly involved in protein binding (72.36% of the 123 annotated peptides were GO 0005488, 89 proteins, **Figure S5A**). Most cellular processes were associated with cellular component organization and biogenesis, and cellular organization (**Figure S5B**). Cellular components ontology is used to provide insights into the cellular regions in which gene products are active; based on the information in this category, 128 of the identified proteins were intercellular, 118 were cytoplasmic, and 125 were associated with vesicles (**Figure 2B**). Moreover, using a protein-protein interaction (PPI) network of the SNAP29-interacting proteins, we identified 41 proteins with a degree of connectivity ≥5. In summary, following OGD, SNAP29-interacting proteins were mainly related to vesicle transportation via PPI in the cellular regions (**Figure 2C**). This result led us to focus on SNAP29 involvement in synaptic function following OGD.

### SNAP29 reduction at synaptic sites significantly suppressed the efficiency of synaptic transmission

**Figure 3:**
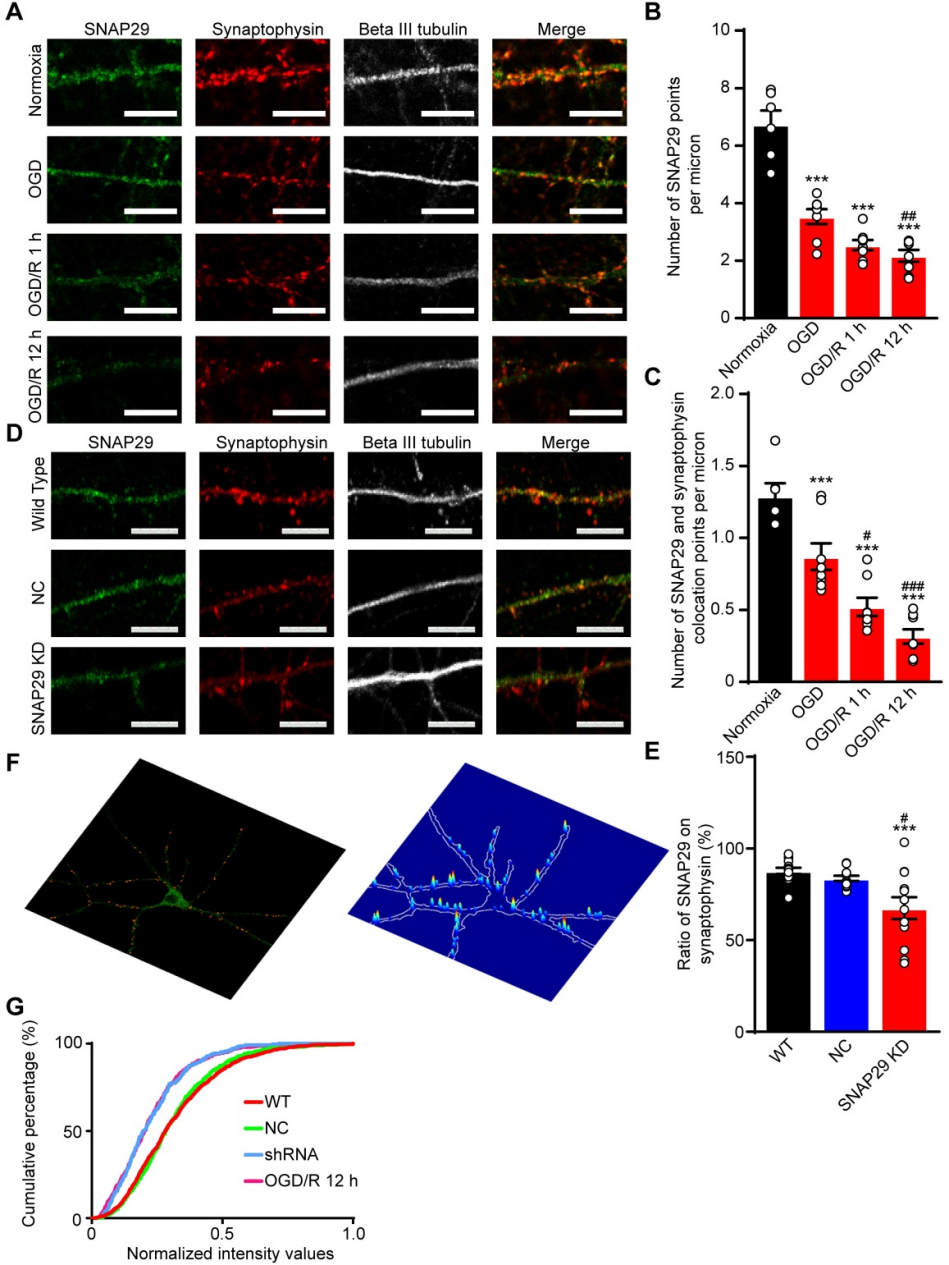
SNAP29 reduction induced by OGD/R exposure or SNAP29 KD significantly decreased the SNAP29 distribution at the synaptic sites. **A.** The colocalization of SNAP29 and synaptophysin was presented in neurons following OGD/R exposure (Scale bar: 10 µm). **B.** The numbers of SNAP29 in per micron were expressed in neurons following OGD/R exposure (***p < 0.001 vs. normoxia group, ^##^*p* < 0.01 vs. OGD group, n = 6 per group, one-way ANOVA; Bonferroni post-test). **C.** The numbers of SNAP29 colocalizing with synaptophysion in per micron were expressed in neurons following OGD/R exposure (*p < 0.05 and ***p < 0.001 vs. normoxia group, ^##^*p* < 0.01 and ^###^*p* < 0.001 vs. OGD group, n = 6-8 per group, one-way ANOVA; Bonferroni post-test) **D.** The colocalization of SNAP29 and synaptophysin was present in neurons after shRNA transfection (Scale bar: 10 µm).** E.** The ratio of SNAP29 on synaptophysin were expressed in neurons after shRNA transfection (***p < 0.001 vs. WT group, ^#^*p* < 0.01 vs. NC group, n = 12-13 per group, one-way ANOVA; Bonferroni post-test). **F.** Typical punctiform signals of synaptophysin puncta of cortical neurons are highlighted (red points in left panel). SNAP29 fluorescence intensity overlaid on a confocal image of the corresponding cortical neurons at presynaptic sites (right panel). **G.** Accumulation index plots for evaluating SNAP29 redistribution by heterogeneity of the fluorescence signal of SNAP29 and synaptophysin along the processes are presented. Data are shown as mean and SEM.

To confirm the bioinformatics analysis results, immunofluorescence staining of SNAP29 and synaptophysin was performed (**Figure 3A**). After OGD and OGD/R, the number of SNAP29 spots decreased, and SNAP29-synaptophysin colocalization was significantly reduced, relative to NC (**Figure 3B-C**). The fact that SNAP29 was reduced in synaptophysin positive presynaptic terminals suggests that SNAP29 may be involved in presynaptic function. To explore the role of SNAP29 in synaptic function, *Snap29* shRNA was used to knock down intrinsic SNAP29 levels in cultured cortical neurons (**Figure S6**; **Figure 3D**). Similar to the effects of OGD, we observed decreased presynaptic distribution of SNAP29 following SNAP29 knockdown (hereafter as SNAP29 KD) at the presynaptic sites localized with synaptophysin (**Figure 3E**). Immunofluorescence staining of PSD95 and synaptophysin was used to observe the synaptic structures. Colocalization of PSD95 and synaptophysin was not influenced by SNAP29 protein levels (**Figure S7A-B**), indicating that SNAP29 knockdown influenced only the distribution of SNAP29 protein at the synapse, but did not damage the synaptic ultrastructure. Further, the presynaptic vesicle pool of SNAP29-overexpressed neurons was also investigated. In the synaptic boutons, the size of the total pool of synaptic vesicles was calculated. Compared with the neurons of the WT and GFP groups, the size of the synaptic vesicle pool was increased in the SNAP29 overexpression group (**Figure S7E-F**). However, the uniform distribution of pre-synaptic vesicles in the SNAP29 over-expressing neurons made it impossible to define the “docked” vesicles, which would have been regarded as the readily releasable pool.

Based on the effect of SNAP29 knockdown at synaptic sites, we asked whether SNAP29 KD mimics SNAP29 reduction following OGD/R 12 h. First, we assessed the SNAP29 distribution along the neuronal processes at the presynaptic sites of cortical neurons, which were indicated by the presynaptic marker synaptophysin. Next, only the colocalized signals of SNAP29 and synaptophysin along the processes were extracted (**Figure S7C**). Then, the intensities of SNAP29 at the colocalized puncta were acquired and normalized with those of synaptophysin (**Figure 3F**). Finally, we assessed SNAP29 accumulation in puncta using an unbiased accumulation index 15 (**Figure 3G**; **Figure S7D**). Reduction of SNAP29, resulting from shRNA knockdown or OGD/R, decreased the SNAP29 accumulation index compared with those of the WT and NC groups, indicating that the SNAP29 distribution decreased at the presynaptic sites. Moreover, shRNA knockdown and OGD/R resulted in similar SNAP29 accumulation indexes, while there was no significant difference between the WT and NC groups (detailed statistical results are shown in **Table 4**). This evidence supports the notion that SNAP29 knockdown with shRNA can mimic the reduction in SNAP29 at the presynaptic site that is observed following OGD/R. Therefore, we subsequently used shRNA knockdown to investigate the effects of SNAP29, because this technique allowed us to exclude the disturbance of other mechanisms that accompany OGD/R.

### SNAP29 reduction disrupted synaptic function in a presynaptic manner

**Figure 4:**
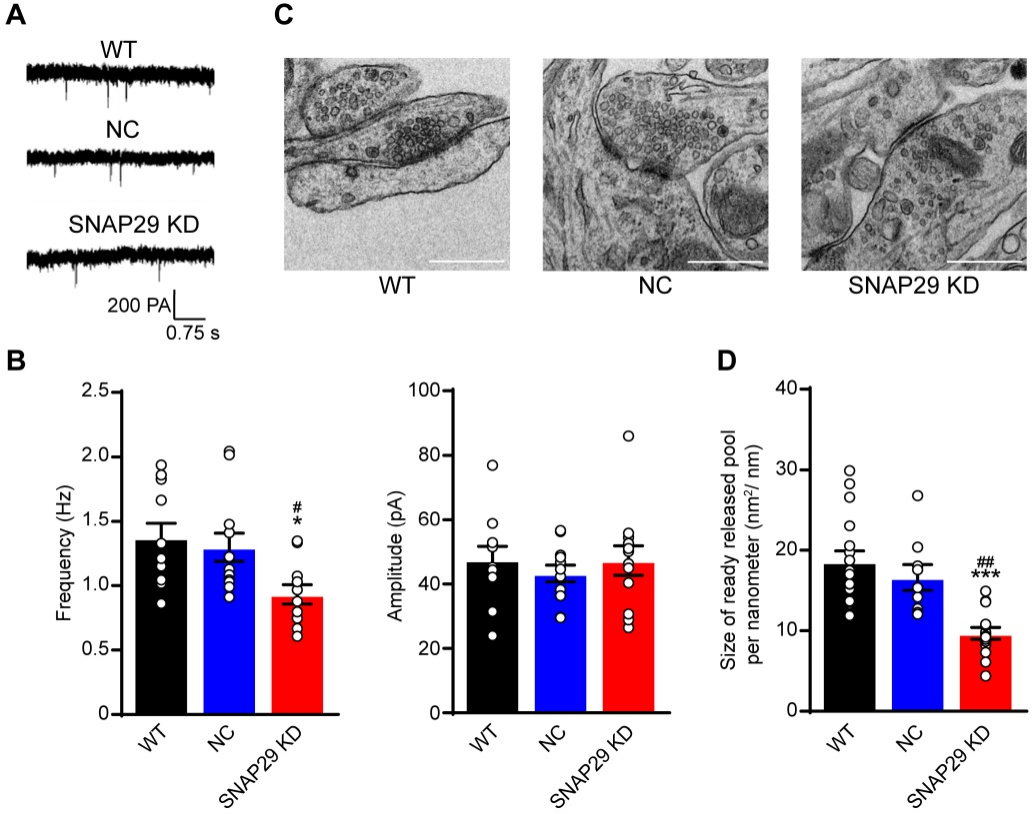
SNAP29 reduction contributed to the synaptic dysfunction of the cortical neurons in the pre-synaptic manner in neurons. **A.** Representative results from whole-cell recording method. **B.** The analysis of frequency and amplitude of AMPA-mediated mEPSCs in neurons after shRNA transfection were present (***p < 0.001 vs. normoxia group, n = 11 per group, one-way ANOVA; Bonferroni post-test). **C.** The images of synaptic ultrastructure of neurons were acquired with TEM in WT, NC and SNAP29 KD groups. **D.** The size of readily releasable pool was calculated (***p < 0.001 vs. WT group, ^##^*p* < 0.01 vs. NC group*,* n = 10-15 per group, one-way ANOVA; Bonferroni post-test)**.** Data are shown as mean and SEM.

Based on our finding that the ratio of SNAP29 to synaptophysin decreased after SNAP29 reduction, we next used whole-cell recording methods to evaluate synaptic function after SNAP29 knockdown with shRNAs (**Figure 4A**). Reductions of SNAP29 significantly reduced the frequency, but not the amplitude, of AMPAR-mediated mEPSCs (**Figure 4B**), suggesting that glutamatergic transmission decreased due to a reduction in presynaptic release. Furthermore, the ultrastructure of the synapses was observed by TEM (**Figure 4C**). Asymmetrical (type I) synapses 46, which are generally regarded as excitatory synapses, were selected for further analysis. SNAP29 reduction resulted in a dispersed distribution of presynaptic vesicles at these asymmetrical synapses (**Figure 4C**) and significantly decreased the size of the readily releasable pool of these vesicles (**Figure 4D**). Although the specific step in the presynaptic vesicle cycle that was impaired by SNAP29 knockdown requires additional exploration, our findings provide further evidence that synaptic dysfunction resulted from dysfunction in presynaptic release.

### SNAP29 reduction disrupted neural functions by impairing long-range neural projections

**Figure 5:**
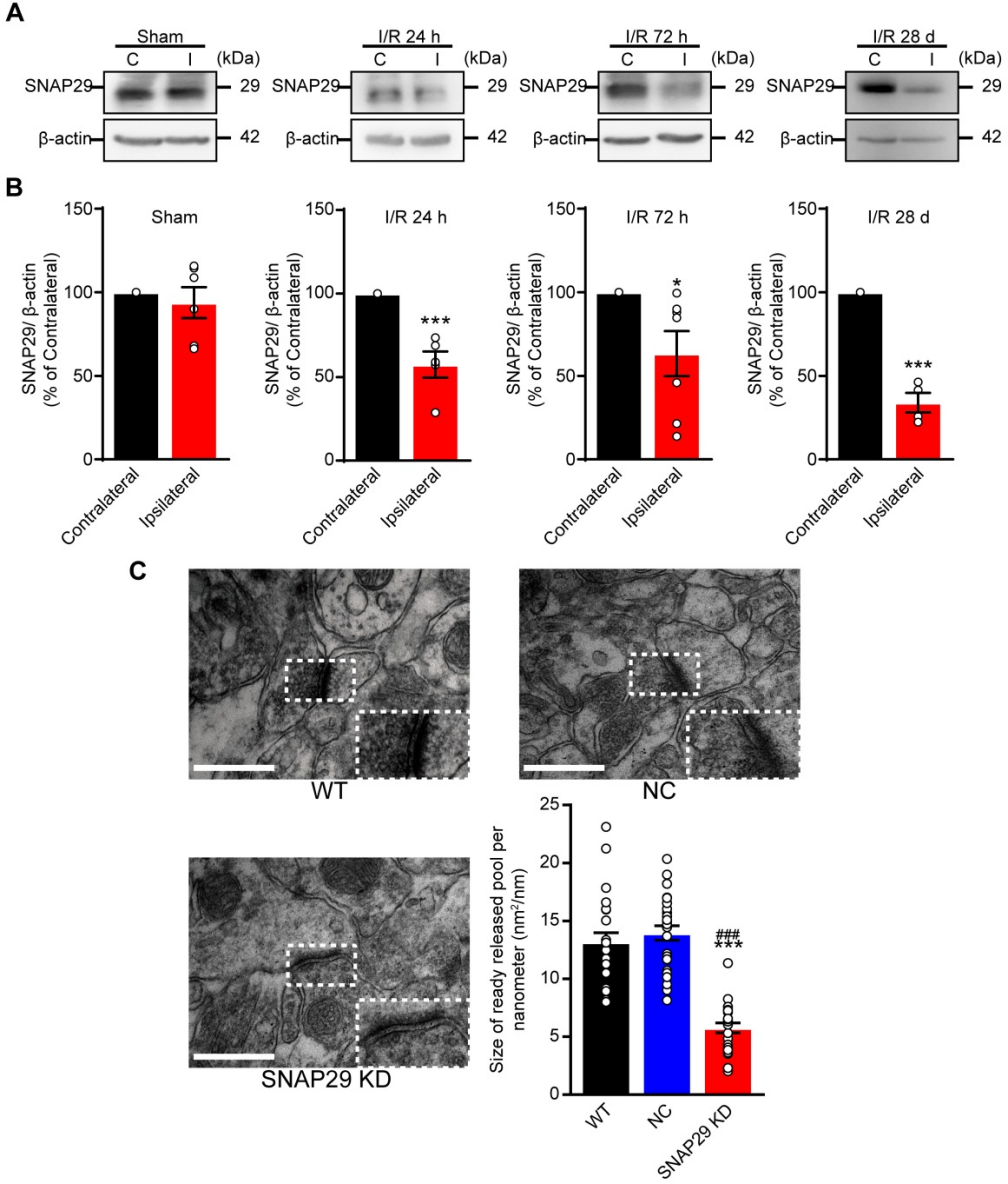
SNAP29 protein levels decreased in the ipsilateral cerebral regions after 1 h of acute ischemia followed by 24 h, 72 h and 28 days reperfusion in MCAO mice. **A.** SNAP29 in ipsilateral (I) or contralateral (C) cerebral regions of MCAO was detected with western blot assay, and quantitative analysis was shown in **B** (*p < 0.05 and ***p < 0.001 vs contralateral cerebral regions*,* n = 6 in Sham, 24 h and 72 h groups, n = 4 in 28 d group, Studentʼs t-test; one-tailed). **C.** The images of synaptic ultrastructure of neurons in brain slices were acquired with TEM in WT, NC and SNAP29 KD groups and the size of readily releasable pool was calculated (***p < 0.001 vs. WT group, ^###^*p* < 0.05*,* vs. NC group, n = 24 per group, one-way ANOVA; Bonferroni post-test). Data are shown as mean and SEM.

Our *in vitro* results revealed that SNAP29 protein levels decreased in cultured cortical neurons following OGD/R, and the reduction in SNAP29 played a vital role in synaptic functions. Therefore, we next determined whether similar effects can be observed in MCAO mice. SNAP29 protein levels were reduced in the penumbra region of MCAO mice, as determined by western blotting (**Figure 5A-B**). Ischemia naturally results in an infarction area and affects nearby vessels, creating a lesion. The hippocampus is the main region affected by ischemia and is important for cognitive function. Therefore, we next evaluated hippocampal structure and function, and associated cognitive impairments. To explore the effect of SNAP29 reduction on* in vivo* neural functions, AAV carrying *Snap29* shRNA were injected into the CA1 region of the hippocampus in WT mice (**Figure S8A**). The synaptic ultrastructures in WT, NC, and SNAP29 KD mice were imaged by TEM (**Figure 5C**). The size of the readily releasable pool of asymmetrical synapses significantly decreased after SNAP29 knock down. Notably, the presynaptic vesicles were also dispersed at the presynaptic sites in SNAP29 KD mice. We therefore concluded that the impaired synaptic transmission efficiency caused by SNAP29 reduction resulted from dysfunction of presynaptic machinery.

**Figure 6:**
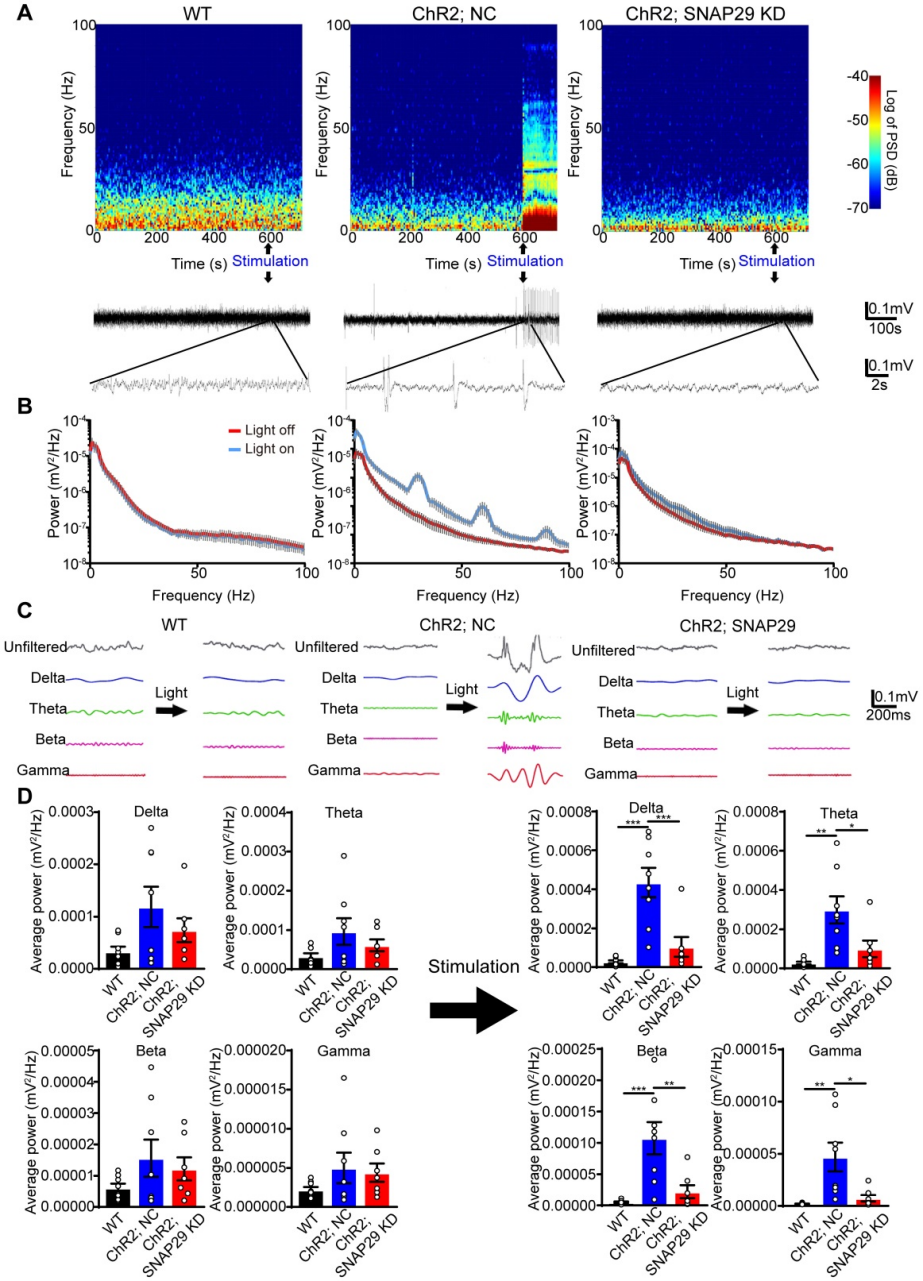
SNAP29 KD in hippocampus impaired the interactions between hippocampus and prefrontal cortex after AAVs simultaneously carrying *Snap29* shRNA and hChR2 channels were injected into the CA1 region of hippocampus. **A.** Example spectrogram (top), raw traces (middle) and traces in different bands (bottom) before and during optogenetic stimulation (blue light) were present. Left panel: WT mouse without AAV transfection. Middle panel: hChR2/NC mouse with AAV transfection of hChR2 channels and shRNA vector. Right panel: hChR2/SNAP29 KD mouse with AAV transfection of hChR2 channels and *Snap29* shRNA. **B.** Power spectra of the local field potential signals in the prefrontal cortex was present in WT, hChR2; NC and hChR2; SNAP29 KD groups. **C.** The delta, theta, beta and gamma band power were present before and during light stimulation in WT, hChR2; NC and hChR2; SNAP29 KD groups, and the power of them was shown in **D** (*p < 0.05, **p < 0.01, and ***p < 0.001, n = 8, one-way ANOVA; Bonferroni post-test). Data are shown as mean and SEM.

We next used optogenetic methods to evaluate whether this dysfunction was accompanied by dysfunction of the neural circuitry. As reduction of SNAP29 in the hippocampus impaired synaptic function, hippocampus-mPFC projection was selected to test our hypothesis. To explore the function of SNAP29 reduction in the neural circuitry (**Figure 6**), we designed an AAV vector that introduced ChR2 and *Snap29* shRNA together into the same neuron (**Figure S8B-D**). After ChR2 protein and *Snap29* shRNA were successfully expressed in hippocampal neurons, optical stimulation with 473 nm light was applied to the hippocampus, and the field potential was recorded in the mPFC (**Figure S8C**). In the NC group, light stimulated the hippocampus-mPFC circuitry, which was taken as evidence for transduction of ChR2 channels. However, when ChR2 channels and *Snap29* shRNAs were transduced simultaneously, the light-stimulated field potentials in the mPFC were blocked, indicating that SNAP29 reduction impaired the neuronal projection from the hippocampus to the mPFC (**Figure 6A-B**). We also measured the powers of the delta, theta, beta, and gamma phases in the mPFC after optical stimulation of the hippocampus (**Figure 6C-D**). Without stimulation, there was no difference in phase power among the three groups (**Figure 6D**, left panel). After stimulation, the powers of all phases increased significantly in the NC group (**Figure 6D**, right panel), which were blocked by SNAP29 knockdown. These results suggest that SNAP29 reduction in cerebral regions might impair communication with multiple regions.

### Deficits in presynaptic SNAP29 critically affect cognitive function

**Figure 7:**
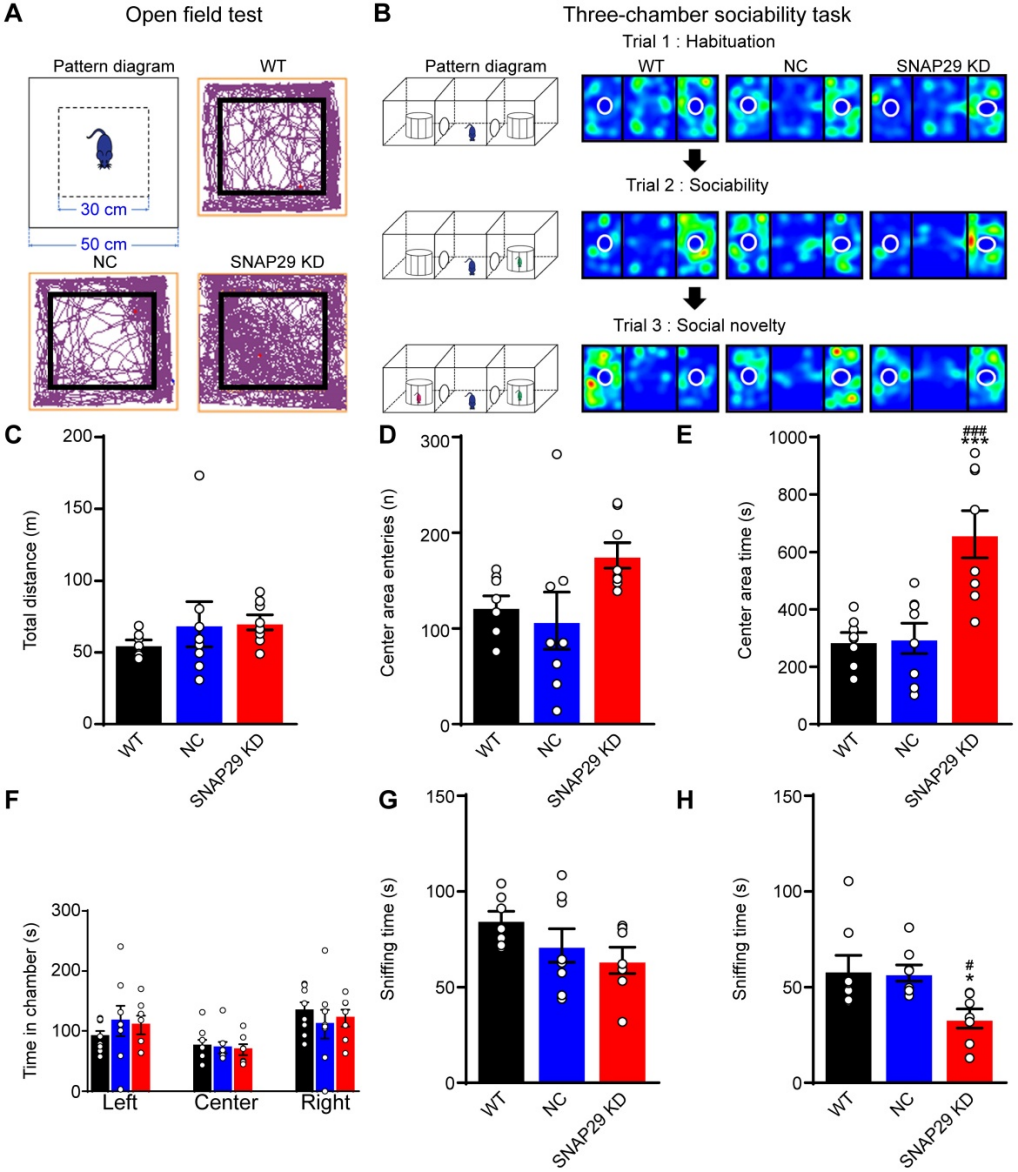
SNAP29 KD in the CA1 hippocampal region increased indifference to danger and caused social dysfunction. **A.** The diagrammatic sketch and representative results from the open field test. **B.** The schematic process diagram and representative results from the three-chamber social test were presented. The total distance traveled, the number of entries into the center and the spent time in the central area of the open field test were shown in **C, D** and** E** (***p < 0.001 vs. WT group, ^###^*p* < 0.001 vs. NC group*,* n = 8 per group, one-way ANOVA; Bonferroni post-test). The time in each chamber (Trial1), sniffing time for strange#1 (Trial 2) and sniffing time for strange#2 (Trial 3) of the three-chamber social test were present in **F, G** and **H**. (*p < 0.001 vs. control group, ^#^*p* < 0.001 vs. NC group*,* n = 8 per group, one-way ANOVA; Bonferroni post-test). Data are shown as mean and SEM.

Since we found that long-range neural projections were impaired after SNAP29 knockdown in the hippocampus, we subsequently asked whether SNAP29 reduction was sufficient to cause cognitive impairment in mice. The effects of loss of function of SNAP29 in the CA1 region were subsequently evaluated using behavioral tests. First, an open field test was carried out in WT, NC, and SNAP29 KD mice (**Figure 7A**). Mice naturally tend to approach a protective wall rather than potentially expose themselves to danger in the center of an open field. Although there were no significant differences in the total traveled distance or number of center area entries among the three experimental groups, the SNAP29 KD mice spent much more time in the center field than the WT and NC mice (**Figure 7C-E**). These augmented exploration activities and suppressed thigmotaxis suggest that SNAP29 knockdown motivated the mice to explore and show indifference to danger. Second, three-chamber sociability tests were conducted to observe social behaviors after SNAP29 knockdown (**Figure 7B**). The time that the mice spent in the different regions (left, center, and right) of the chamber was not significantly different among the groups (**Figure 7F**). Moreover, in the sociability session (**Figure 7G**), there were no differences in the time that the mice spent sniffing stranger #1 among the three groups, indicating that social actions were not significantly impaired. However, in the social novelty session (**Figure 7H**), the SNAP29 KD mice spent less time sniffing stranger#2 than the WT mice and NC mice did, suggesting that the SNAP29 KD mice may experience social memory dysfunction.

**Figure 8:**
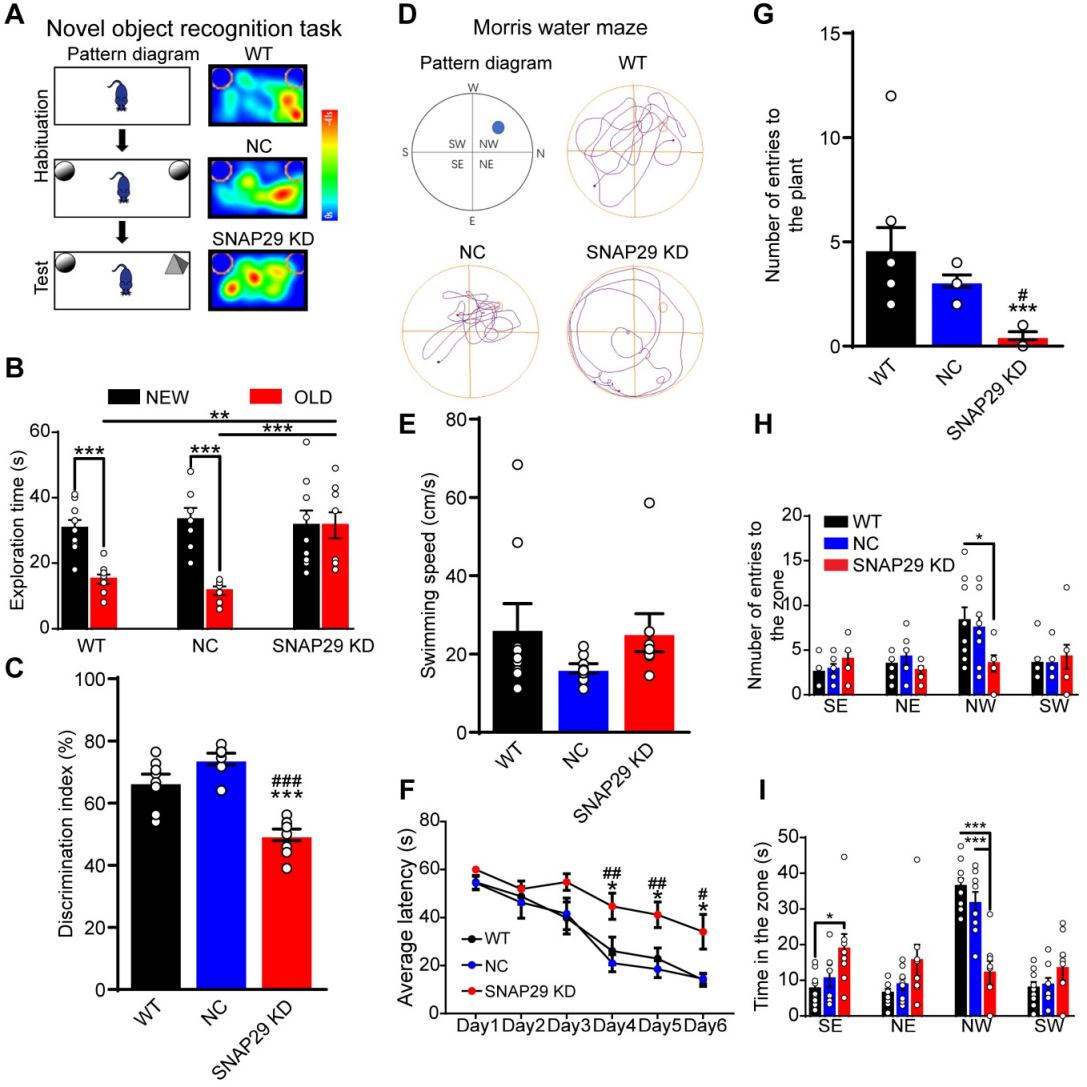
SNAP29 KD in the CA1 hippocampal region resulted in the memory defects in mice. **A.** A schematic of the novel object recognition test procedures and representative results were shown. **B.** The exploration time was present in WT, NC and SNAP29 KD groups (***p < 0.001 and **p < 0.01*,* n = 8 per group, one-way ANOVA; Bonferroni post-test).** C.** The discrimination indexes were present in WT, NC and SNAP29 KD groups (***p < 0.001 vs. WT group, ^###^*p* < 0.001 vs. NC group*,* n = 8 per group, one-way ANOVA; Bonferroni post-test). **D.** The schematic and representative navigation traces on Day 7 in the Morris water maze test were shown. **E.** The swimming speed was present. **F.** The average escape latencies were present in WT, NC and SNAP29 KD groups (*p < 0.05 vs. WT group, ^##^*p* < 0.01 and ^#^*p* < 0.05 vs. NC group*,* n = 8-9 per group, one-way ANOVA; Bonferroni post-test). **G, H** and **I.** The number of entries to the plant zone, the number of entries to each quadrant, and the duration in each quadrant were presented in WT, NC and SNAP29 KD groups (***p < 0.001*,* n = 8-9 per group, one-way ANOVA; Bonferroni post-test). Data are shown as mean and SEM.

Next, we evaluated the possible changes in memory and learning induced by SNAP29 knockdown. First, a novel object recognition test was used to assess short-term memory by assessing the ability of the mice to remember whether they had previously encountered a particular object (**Figure 8A**). Generally, mice will spend more time observing and exploring a novel object than a familiar one. During the testing period, SNAP29 KD mice spent more time on the familiar object than the WT and NC mice did (**Figure 8B**). Moreover, the discrimination index was significantly lower for the SNAP29 KD mice (**Figure 8C**). Second, a Morris water maze was used to further explore memory function in the SNAP29 KD mice (**Figure 8D**). On the first day of the spatial navigation trials, there was no difference in the swimming speed among the groups (**Figure 8E**). However, during training days 4-6, SNAP29 KD mice exhibited longer escape latencies than the WT and NC mice (**Figure 8F**). When the platform was removed, analysis of the navigation paths showed that entry time to the original platform position, number of entries, and time spent in the target quadrant decreased in the SNAP29 KD mice compared with the other two groups (**Figure 8G-I**). These results suggest that SNAP29 knockdown markedly impaired cognitive and memory functions in mice.

## Discussion

Poststroke cognitive dysfunctions imply synaptic dysfunction. In this study, the protein levels of SNAP29 decreased immediately after ischemia and were sustained at low levels. Notably, the reduction in SNAP29 did not influence autophagic flux, but did influence synaptic transmission efficiency following acute ischemia and during subsequent reperfusion. These findings have several important implications. First, synaptic dysfunction occurred before autophagy following ischemic stress. Second, this study provides evidence that SNAP29 protein levels do not contribute to neuronal viability but rather to synaptic function, which is different from a previous report that SNAP29 is a key protein for neuronal health, including tissue development and homeostasis 17. Still, the role of SNAP29 in neuronal phagocytotic death was not fully addressed in our study. So, we cannot exclude the possibility that the changed protein level of SNAP29 contributed to phagocytotic death, which is associated with neuropathologies and deserves further investigation. Deficiencies or injuries in synaptic function are associated with a wide range of brain disorders and cognitive impairments, including neurodegenerative and psychiatric diseases such as poststroke dementia. A hallmark of synaptic specializations is their dependence on highly organized complexes of proteins that interact with each other. Therefore, the loss or modification of key synaptic proteins might directly affect the properties of such networks and, ultimately, cognitive functions 47. Under ischemic insults, the bioinformatics analysis of SNAP29-interacting differentially expressed proteins suggests that ischemia-induced SNAP29 reduction may occur at synapses. Consistent with our study result that SNAP29 protein levels did not influence the number of synapses, Pan et al. reported that SNAP29 does not affect synapse density 48. However, SNAP29 knock down in the CA1 region of the hippocampus did influence basal synaptic transmission, which is different from the results of Pan et al. and Su et al. who reported that SNAP29 functions as a regulator of SNARE complex disassembly and contributes to postfusion recycling of SNARE components with acute injection of SNAP29 into presynaptic neurons 27. Thus, those authors considered SNAP29 to be an activity-dependent negative modulator of synaptic transmission whereas our study indicates that SNAP29 is a positive modulator. In our opinion, this difference may be a result of the different SNAP29 manipulation methods. So, a method for comparing these methods is required. Additionally, when SNAP29 was overexpressed in neurons, the size of the presynaptic vesicle pool increased significantly. However, it was difficult to determine the readily releasable pool. To summarize, the number of presynaptic vesicles increased following SNAP29 overexpression. Based on the vital role of SNAP29 in autophagic machinery and synaptic vesicle cycle 19, 27, 48, 49, the detailed mechanism of this phenomenon deserves thorough investigation.

In our study, we found that SNAP29 reduction in hippocampal neurons influenced long-range functional connections the mPFC. Multiple trace theory posits that the hippocampus is always associated with the storage and retrieval of detailed episodic information of a memory 50, 51. Subsequently, schematic information of this memory is constructed in the cortex. The long-range pathway from the hippocampus plays an essential role in modulating learning-memory processing and psychiatric disorders 52, 53. The hippocampus and the prefrontal cortex anatomically connect both directly and indirectly manner 54, 55. The circuit between the hippocampus and the mPFC is important for learning and memory processes. In this circuit, it is generally believed that the hippocampus plays a crucial role in spatial and temporal contextual memory 43, 56, and that the mPFC is related to memory retrieval 57, 58, while some functions, such as spatial memory consolidation, rely on connections to additional regions 59. Our results revealed that neurons in the hippocampus form functional synapses with those in the mPFC, so the hippocampus can directly alter activity via projections to regions such as the prefrontal cortex. Nevertheless, we cannot exclude the possibility that hippocampal dysfunction can modulate learning-memory processing via multiple parallel pathways. Also, the affected cognitive functions will not be confined to those we report here. In this study, the lesion region in the bilateral hippocampus was simulated. In future study, the symptoms will be different if the lesion is restricted to one side of the hippocampus or is restricted to the prefrontal cortex, thalamus, or other cerebral tissues.

An active area of research is how changes in oscillatory synchrony under a variety of experimental conditions can influence network dynamics. Generally, increased oscillatory synchrony indicates enhanced neural communication 60. These neuronal oscillations can be separated into different frequency bands. For example, delta oscillations (0.5-3 Hz) have been shown to be involved in sleep 61; theta oscillations (3.5-7 Hz) in cognitive functions 23; beta oscillations (13-30 Hz) in sensorimotor processing, attention, and emotion; and gamma oscillations in the process of memory encoding 62, 63. After transfection of the hippocampus with ChR2, light stimulation increased delta, theta, beta, and gamma powers in the mPFC. However, when ChR2 and SNAP29 shRNA were expressed simultaneously in the hippocampus, responses to light stimulation in the mPFC were significantly decreased. Since the power of all the frequency bands stimulated by ChR2 activation in the mPFC decreased after SNAP29 knockdown, we conclude that the connectivity between the hippocampus and the mPFC was decreased. Our results, therefore, expand our knowledge of the hippocampus-prefrontal cortex neural circuits underlying learning-memory modulation and pave the way for further dissection of SNAP29-involved circuit-level mechanisms in different types of neurons and between different cerebral regions after acute ischemic stroke.

In aging populations, the prevalence and incidence of cerebrovascular diseases continue to increase year by year, together with the number of individuals with cognitive impairment. Emerging clinical studies have examined the significance and features of poststroke cognitive impairment 64. A nationwide Korean study defined poststroke cognitive impairment as any major cognitive dysfunction occurring more than 3 months after a stroke without the presence of prestroke cognitive dysfunction 65. However, Akiyemi et al. found that poststroke dementia has different neurodegenerative characteristics in hippocampal Alzheimer pathology compared with other dementias 66, indicating that poststroke dementia may not share the same mechanisms as Alzheimer's disease and requires independent exploration. Although we found that ischemia-induced SNAP29 reduction was closely associated with poststroke cognitive impairment, there is still a lack of information regarding SNAP29 protein levels in other neurodegenerative diseases. To confirm whether poststroke cognitive dysfunction shares pathways with other neurodegenerative diseases, SNAP29-involved pathology in other neurodegenerative diseases needs to be investigated. Additionally, in this study we only provide results in male mice; therefore, the role of SNAP29 in neural circuits after ischemic injury in female mice should be explored.

Clinically, poststroke cognitive impairment or dementia is mainly defined as dementia arising within three months after the occurrence of stroke 67. Meanwhile, some poststroke cognitive impairments or dementia occur beyond three months or after recurrent stroke(s). Determination of cognitive impairment at the acute stage after stroke may provide a vital indication to the clinician for early cognitive rehabilitation 68. In recent years, any strategy that alleviates vascular disease is considered preventative for post-stroke cognitive impairment 69. Although concomitant usage of aspirin and dipyridamole; concomitant usage of antihypertensives, antithrombotic agents, and lipid-lowering drugs; physical activity; and healthy diet are all regarded as efficient treatments against poststroke cognitive impairment 70, 71, the target options for post-stroke cognitive impairment are still limited and their effects are modest 67. The role of SNAP29 has gradually been recognized, including in autophagy machinery and synaptic plasticity; however, SNAP29-targeted therapies have rarely been reported. In this study, the expression level of SNAP29 decreased after acute ischemia and this reduction was significant in neurons. Thus, method to target the expression or function of SNAP29 is a prospective area for further exploration. Debora et al. reported that NIMA-never in mitosis gene A-related kinase 3 (NEK3) modulates the membrane association of SNAP29 via phosphorylation at serine 105, suggesting that pharmacological induction of NEK3 may improve the function of SNAP29 after stroke 72. Additionally, exogenous SNAP29 administration via intravenous vectors, such as HIV TAT-mediated protein transduction (in which the TAT motif facilitates BBB penetration 73) deserves exploration.

In summary, we revealed that SNAP29 protein levels in neurons decreased after ischemic stress. This SNAP29 reduction disrupted synaptic homeostasis and neural circuits. Further, SNAP29 reduction emerged as a causative factor of cognitive impairment. So, strategies to maintain the function of SNAP29 may be vital for mitigating poststroke cognitive impairment. Our study not only reveals a new target for improving cognitive functions after ischemic stroke, but also supports a novel pathology for neurodegenerative disorders.

## Supplementary Material

Supplementary figures.

## Figures and Tables

**Table 1:** The list of antibodies used in the current western blotting (WB) and immunofluorescence (IF) studies

Antibodies and Reagents	Type	Source/cat. #	Dilution (WB)	Dilution (IF)
LC3B	Rabbit pAb, IgG	Proteintech [14600-1-AP]	1:1000	N/A
P62	Rabbit pAb, IgG	Abcam [ab109012]	1:1000	N/A
SNAP29	Rabbit pAb, IgG	Proteintech [12704-1-AP]	1:1000	N/A
β-actin	Mouse mAb, IgM	Proteintech [60008-1-IG]	1:3000	N/A
SNAP29	Rabbit mAb, IgG	Abcam [ab181151]	N/A	1:200
PSD95	Rabbit pAb, IgG	CST [2507s]	N/A	1:100
Synaptophysin	Mouse mAb, IgG	Abcam [ab8049]	N/A	1:200
β-III tubulin	Chicken pAb, IgY	Abcam [ab41489]	N/A	1:800
Goat anti-Rabbit IgG (H+L) Highly Cross-Adsorbed Secondary Antibody-Oregon Green 488	Goat pAb, IgG	Thermo Fisher [O-11038]	N/A	1:400
Goat anti-Rabbit IgG (H+L) Highly Cross-Adsorbed Secondary Antibody-Alexa Fluor Plus 647	Goat pAb, IgG	Thermo Fisher [A32733]	N/A	1:400
Goat anti-Mouse IgG (H+L) Highly Cross-Adsorbed Secondary Antibody, Alexa Fluor Plus 647	Goat pAb, IgG	Thermo Fisher [A32728]	N/A	1:400
Goat Anti-Chicken IgY H&L (Alexa Fluor 594)	Goat pAb, IgY	Abcam [ab150176]	N/A	1:400

**Table 2:** The list of reagents and consumables used in the current study

Reagent	Brand	Cat.
RIPA	Cell Signal Technology	9806S
Revert Aid First Strand cDNA Synthesis Kit	ThermoFisher Scientific	K1622
PowerUp™ SYBR™ Green Master Mix	ThermoFisher Scientific	A25742
DAPI	SouthernBiotech	0100-29
Poly-vinylidene difluoride membranes	Healthcare Life science	0.2 μm PVDF
Pierce™ BCA Protein Assay Kit	ThermoFisher Scientific	23225
No glucose DMEM	ThermoFisher Scientific	11966025
CCK-8	Beyotime Biotechnology	C0038
Silicone thread embolism	RWD Life Science	970-00091-00
Lactate dehydrogenase assay kit	abcam	Ab102526
Bicuculline	Sigma-Aldrich	S2694-5 MG
D(-)-2-Amino-5-phosphonopentanoic acid	MedChemEpress	HY-100714A
TTX	Sigma-Aldrich	A3109
Thick‐walled boro‐silicate glass	VWR Scientific	N/A
DMEM, high glucose, Pyruvate	ThermoFisher Scientific	11995065
Neurobasal^TM^-A Medium	ThermoFisher Scientific	11888022
B-27^TM^ Supplement (50×), serum free	ThermoFisher Scientific	17504044
Calcium chloride	Sigma-Aldrich	C4901-100 G
Magnesium chloride	Sigma-Aldrich	M8266-100 G
D(-)-2-Amino-5-phosphonopentanoic acid	Sigma-Aldrich	A8054-1 MG
HBSS-Hank's, calcium, magnesium, no phenol red	ThermoFisher Scientific	14025076
Trypsin-EDTA (0.25%), phenol red	ThermoFisher Scientific	25200056
MG132	MedChemExpress	HY-13259
PhosSTOP EASYpack; Phosphatase Inhibitor Cocktail Tablets Supplied in Foil Blister Packs	Roche	04906845001
Protease Inhibitor Cocktail	Sigma-Aldrich	P8340-1 ml
Glycine	Sigma-Aldrich	V900114-500 G
Sodium dodecyl sulfate	Sigma-Aldrich	L5750-1 KG
Acrylamide	Sigma-Aldrich	V900845-1 KG
Tris base	Sigma-Aldrich	10708976001-1 KG
Sodium chloride	Gong Ke Ji You Xian Co.,Ltd	N/A
Glutaraldehyde solution (50%)	Sinopharm Chemical Reagent Co.,Ltd	30092595-250 ML
N,N,N′,N′-Tetramethylethylenediamine	Sigma-Aldrich	T7024-25 ML
Ammonium persulfate	Sigma-Aldrich	A9154-100 G
PBS Buffer powder	Zhong Shan Jin Qiao Co.,Ltd	ZLI-9061
Non-fat milk powder	Pu Li Lai Co.,Ltd	P1622
TWEEN® 20	Sigma-Aldrich	V900548-500 ML
Paraformaldehyde	Sigma-Aldrich	16005-1 KG-R
Adenosine 5′-triphosphate disodium salt solution	Sigma-Aldrich	A6559-25 UMO
Ethylene glycol-bis(2-aminoethylether)-N,N,N′,N′-tetraacetic acid	Sigma-Aldrich	E0396-10 G
Potassium chloride	Bei Jing Shi Ji	N/A
6 Well Cell Culture Plate	Sigma-Aldrich	3516
24 Well Cell Culture Plate	Sigma-Aldrich	3524
96 Well Cell Culture Plate	Sigma-Aldrich	3599
Triton X-100	Zhong Shan Jin Qiao Co.,Ltd	ZLI-9308
Fetal Bovine Serum, qualified, heat inactivated, Australia	ThermoFisher Scientific	10100147
Fetal Equine Serum	Solarbio Life Science	S9050-200
Poly-D-lysine hydrobromide	Sigma-Aldrich	P1149-500 MG
Microscope Cover Glass	ThermoFisher Scientific	12-545-80 12CIR.-1
Cell Strainer 70 μm Nylon	Corning	352350
15 ml Polypropylene Conical Centrifuge Tube	R&D	KG2611
50 ml Polypropylene Conical Centrifuge Tube	R&D	KG2811
PageRuler™ Prestained Protein Ladder	ThermoFisher Scientific	26616

**Table 3:** The list of primers used in the current study

ID	Sense primer	Antisense primer
*Snap29*	5'-CACACGGAGAAGATGGTGGACAAG-3'	5'-TTCTGCTCAGGTGGAGGCTCTAC-3'
*β-actin*	5'-TTCGCCATGGATGACGATATC-3'	5'-TAGGAGTCCTTCTGACCCATAC-3'

**Table 4:** The cumulative frequency distribution of SNAP29 was statistically analyzed to reflect SNAP29 expression under different conditions. The differences between groups were statistically analyzed with the Kolmogorov-Smirnov test, and a *p* value < 0.05 was considered significant

Kolmogorov-Smirnov test	*p* value	Significant?
WT vs. NC	0.4679	No
WT vs. OGD/R 1 h	1.7239×10^-33^	Yes
WT vs. SNAP29 KD	8.9275×10^-21^	Yes
NC vs. OGD/R 1 h	3.8896×10^-34^	Yes
NC vs. SNAP29 KD	2.5523×10^-16^	Yes
OGD/R 1 h vs. SNAP29 KD	0.7924	No

**Table 5:** The list of shRNAs used in the current study

Label	Gene ID	TargetSeq	Sequence
pLKD-U6-shRNA (*Snap29*)	NM_053810	5'-GGAAATCGAGGAGCAGGAT-3'	5'-CCGGGGAAATCGAGGAGCAGGATTTCAAGAGAATCCTGCTCCTCGATTTCCTTTTTTG-3'
pAAV-CMV-RFP-U6-shRNA (*Snap29*)	NM_023348.4	5'-GGGAATGCAGACAGAAATTGA-3'	5'-CCGGGCAGATTGAAAGAAGCCATTTCAAGAGAATGGCTTCTTTCAATCTGCTTTTTTG-3'
pAAV-CMV-hChR2(H123R)-mCherry-U6-shRNA (*Snap29* knockdown)	NM_023348.4	5'-GCAGATTGAAAGAAGCCAT-3'	5'-GATCCGGGAATGCAGACAGAAATTGATTCAAGAGATCAATTTCTGTCTGCATTCCCTTTTTTA-3'

## Abbreviations

AAV: adeno-associated viruses
AMPARs-mediated mEPSCs: α-amino-3-hydroxy-5-methyl-4-isoxazole-propionicacid receptor-mediated miniature excitatory postsynaptic currents
APOE: apolipoprotein
BACE1: β-secretase 1
CCK: cell counting kit
DMEM: dulbecco's modified eagle medium
ChR2: channelrhodopsin-2
ChR2, NC: mouse with AAV transfection of ChR2 and negative virus
ChR2, SNAP29 KD: mouse with AAV transfection of ChR2 and SNAP29 shRNA
co-IP: co-immunoprecipitation
EGTA: Ethylenebis (oxyethylenenitrilo) tetraacetic acid
GABAA receptors: the type A receptors for γ-aminobutyric acid
GO: Gene Ontology
IACUC: Institutional Animal Care and Use Committee
LC3: microtubule-associated protein 1 light chain 3
LDH: lactate dehydrogenase
CO-IP: Co-immunoprecipitation
LC-MS: liquid chromatograph-mass spectrometer
MCAO: multiple cerebral artery occlusion
mPFC: medial prefrontal cortex
NC: negative control
NMDA receptor: N-methyl-D-aspartic acid receptor
OGD: oxygen-glucose deprivation
OGD/R: oxygen-glucose deprivation/reperfusion
PPI: protein-protein interaction
qPCR: real-time Quantitative polymerase chain reaction
RRP: readily releasable pool
SIM: Structure Illumination Microscopy
sRAGE: soluble receptor for advanced glycation end-products
soluble N-ethylmaleimide-sensitive factor receptor): R-SNAREs
SNAP25: synaptosomal-associated protein 25
SNAP29: Synaptosome Associated Protein 29
SNAP29 KD: SNAP29 knockdown
Stx17: syntaxin 17
TEM: Transmission Electron Microscope
VAMP8: vesicle associated member protein 8
WT: wild type

## References

[1] Feigin VL, Norrving B, Mensah GA (2017). Global Burden of Stroke. Circ Res.

[2] Tatemichi TK, Desmond DW, Stern Y, Paik M, Sano M, Bagiella E (1994). Cognitive impairment after stroke: frequency, patterns, and relationship to functional abilities. J Neurol Neurosurg Psychiatry.

[3] Mellon L, Brewer L, Hall P, Horgan F, Williams D, Hickey A (2015). Cognitive impairment six months after ischaemic stroke: a profile from the ASPIRE-S study. BMC Neurol.

[4] Nys GM, van Zandvoort MJ, van der Worp HB, de Haan EH, de Kort PL, Jansen BP (2006). Early cognitive impairment predicts long-term depressive symptoms and quality of life after stroke. J Neurol Sci.

[5] Sachdev PS, Chen X, Brodaty H, Thompson C, Altendorf A, Wen W (2009). The determinants and longitudinal course of post-stroke mild cognitive impairment. J Int Neuropsychol Soc.

[6] Pendlebury ST, Rothwell PM (2009). Prevalence, incidence, and factors associated with pre-stroke and post-stroke dementia: a systematic review and meta-analysis. Lancet Neurol.

[7] Qian L, Ding L, Cheng L, Zhu X, Zhao H, Jin J (2012). Early biomarkers for post-stroke cognitive impairment. J Neurol.

[8] Kay AM, Simpson CL, Stewart JA Jr (2016). The Role of AGE/RAGE Signaling in Diabetes-Mediated Vascular Calcification. J Diabetes Res.

[9] Zhang T, Yan W, Li Q, Fu J, Liu K, Jia W (2011). 3-n-Butylphthalide (NBP) attenuated neuronal autophagy and amyloid-beta expression in diabetic mice subjected to brain ischemia. Neurol Res.

[10] Sun X, He G, Qing H, Zhou W, Dobie F, Cai F (2006). Hypoxia facilitates Alzheimer's disease pathogenesis by up-regulating BACE1 gene expression. Proc Natl Acad Sci U S A.

[11] Ballard CG, Morris CM, Rao H, O'Brien JT, Barber R, Stephens S (2004). APOE epsilon4 and cognitive decline in older stroke patients with early cognitive impairment. Neurology.

[12] Giltay EJ, van Reedt Dortland AK, Nissinen A, Giampaoli S, van Veen T, Zitman FG (2009). Serum cholesterol, apolipoprotein E genotype and depressive symptoms in elderly European men: the FINE study. J Affect Disord.

[13] Zhang T, Wang H, Li Q, Huang J, Sun X (2014). Modulating autophagy affects neuroamyloidogenesis in an in vitro ischemic stroke model. Neuroscience.

[14] Gold AB, Herrmann N, Swardfager W, Black SE, Aviv RI, Tennen G (2011). The relationship between indoleamine 2,3-dioxygenase activity and post-stroke cognitive impairment. J Neuroinflammation.

[15] Klimkowicz A, Slowik A, Dziedzic T, Polczyk R, Szczudlik A (2005). Post-stroke dementia is associated with alpha(1)-antichymotrypsin polymorphism. J Neurol Sci.

[16] Swardfager W, Winer DA, Herrmann N, Winer S, Lanctot KL (2013). Interleukin-17 in post-stroke neurodegeneration. Neurosci Biobehav Rev.

[17] Mastrodonato V, Morelli E, Vaccari T (2018). How to use a multipurpose SNARE: The emerging role of Snap29 in cellular health. Cell Stress.

[18] Rotem-Yehudar R, Galperin E, Horowitz M (2001). Association of insulin-like growth factor 1 receptor with EHD1 and SNAP29. J Biol Chem.

[19] Morelli E, Ginefra P, Mastrodonato V, Beznoussenko GV, Rusten TE, Bilder D (2014). Multiple functions of the SNARE protein Snap29 in autophagy, endocytic, and exocytic trafficking during epithelial formation in Drosophila. Autophagy.

[20] Wang C, Wang H, Zhang D, Luo W, Liu R, Xu D (2018). Phosphorylation of ULK1 affects autophagosome fusion and links chaperone-mediated autophagy to macroautophagy. Nat Commun.

[21] Mizushima N, Komatsu M (2011). Autophagy: renovation of cells and tissues. Cell.

[22] Itakura E, Kishi-Itakura C, Mizushima N (2012). The hairpin-type tail-anchored SNARE syntaxin 17 targets to autophagosomes for fusion with endosomes/lysosomes. Cell.

[23] Diao J, Liu R, Rong Y, Zhao M, Zhang J, Lai Y (2015). ATG14 promotes membrane tethering and fusion of autophagosomes to endolysosomes. Nature.

[24] Kimura T, Jia J, Kumar S, Choi SW, Gu Y, Mudd M (2017). Dedicated SNAREs and specialized TRIM cargo receptors mediate secretory autophagy. EMBO J.

[25] Dupont N, Jiang S, Pilli M, Ornatowski W, Bhattacharya D, Deretic V (2011). Autophagy-based unconventional secretory pathway for extracellular delivery of IL-1beta. EMBO J.

[26] Zhang M, Kenny SJ, Ge L, Xu K, Schekman R (2015). Translocation of interleukin-1beta into a vesicle intermediate in autophagy-mediated secretion. Elife.

[27] Su Q, Mochida S, Tian JH, Mehta R, Sheng ZH (2001). SNAP-29: a general SNARE protein that inhibits SNARE disassembly and is implicated in synaptic transmission. Proc Natl Acad Sci U S A.

[28] Forsyth JK, Nachun D, Gandal MJ, Geschwind DH, Anderson AE, Coppola G, et al. Synaptic and Gene Regulatory Mechanisms in Schizophrenia, Autism, and 22q11.2 Copy Number Variant-Mediated Risk for Neuropsychiatric Disorders. Biol Psychiatry. 2019 10.1016/j.biopsych.2019.06.029 PMC6925326 31500805

[29] Fuchs-Telem D, Stewart H, Rapaport D, Nousbeck J, Gat A, Gini M (2011). CEDNIK syndrome results from loss-of-function mutations in SNAP29. Br J Dermatol.

[30] Mastrodonato V, Beznoussenko G, Mironov A, Ferrari L, Deflorian G, Vaccari T (2019). A genetic model of CEDNIK syndrome in zebrafish highlights the role of the SNARE protein Snap29 in neuromotor and epidermal development. Sci Rep.

[31] Hamos JE, DeGennaro LJ, Drachman DA (1989). Synaptic loss in Alzheimer's disease and other dementias. Neurology.

[32] Nobili A, Latagliata EC, Viscomi MT, Cavallucci V, Cutuli D, Giacovazzo G (2017). Dopamine neuronal loss contributes to memory and reward dysfunction in a model of Alzheimer's disease. Nat Commun.

[33] Cui D, Sun D, Wang X, Yi L, Kulikowicz E, Reyes M (2017). Impaired autophagosome clearance contributes to neuronal death in a piglet model of neonatal hypoxic-ischemic encephalopathy. Cell Death Dis.

[34] Liu YY, Zhang TY, Xue X, Liu DM, Zhang HT, Yuan LL (2017). Pseudoginsenoside-F11 attenuates cerebral ischemic injury by alleviating autophagic/lysosomal defects. CNS Neurosci Ther.

[35] Yang W, Xie J, Qiang Q, Li L, Lin X, Ren Y (2019). Gedunin Degrades Aggregates of Mutant Huntingtin Protein and Intranuclear Inclusions via the Proteasomal Pathway in Neurons and Fibroblasts from Patients with Huntington's Disease. Neuroscience Bulletin.

[36] Hurn PD, Macrae IM (2000). Estrogen as a neuroprotectant in stroke. J Cereb Blood Flow Metab.

[37] Robison LS, Gannon OJ, Salinero AE, Zuloaga KL (2019). Contributions of sex to cerebrovascular function and pathology. Brain Res.

[38] Zhang X, Tang X, Liu K, Hamblin MH, Yin KJ (2017). Long Noncoding RNA Malat1 Regulates Cerebrovascular Pathologies in Ischemic Stroke. J Neurosci.

[39] Forner S, Martini AC, Prieto GA, Dang CT, Rodriguez-Ortiz CJ, Reyes-Ruiz JM (2019). Intra- and extracellular beta-amyloid overexpression via adeno-associated virus-mediated gene transfer impairs memory and synaptic plasticity in the hippocampus. Sci Rep.

[40] McKenzie BA, Mamik MK, Saito LB, Boghozian R, Monaco MC, Major EO (2018). Caspase-1 inhibition prevents glial inflammasome activation and pyroptosis in models of multiple sclerosis. Proc Natl Acad Sci U S A.

[41] Jullie D, Stoeber M, Sibarita JB, Zieger HL, Bartol TM, Arttamangkul S (2019). A Discrete Presynaptic Vesicle Cycle for Neuromodulator Receptors. Neuron.

[42] Zhao J, Liu X, Huo C, Zhao T, Ye H (2018). Abnormalities in Prefrontal Cortical Gene Expression Profiles Relevant to Schizophrenia in MK-801-Exposed C57BL/6 Mice. Neuroscience.

[43] Tuscher JJ, Taxier LR, Fortress AM, Frick KM (2018). Chemogenetic inactivation of the dorsal hippocampus and medial prefrontal cortex, individually and concurrently, impairs object recognition and spatial memory consolidation in female mice. Neurobiol Learn Mem.

[44] Nakazawa H, Suzuki Y, Ishikawa Y, Bando Y, Yoshida S, Shiosaka S (2019). Impaired social discrimination behavior despite normal social approach by kallikrein-related peptidase 8 knockout mouse. Neurobiol Learn Mem.

[45] Vorhees CV, Williams MT (2006). Morris water maze: procedures for assessing spatial and related forms of learning and memory. Nat Protoc.

[46] Eccles J Fau, Llinas R Fau, Sasaki K (1964). Golgi Cell Inhibition in the Cerebellar Cortex. Nature.

[47] Antonucci F, Corradini I, Fossati G, Tomasoni R, Menna E, Matteoli M (2016). SNAP-25, a Known Presynaptic Protein with Emerging Postsynaptic Functions. Front Synaptic Neurosci.

[48] Pan PY, Cai Q, Lin L, Lu PH, Duan S, Sheng ZH (2005). SNAP-29-mediated modulation of synaptic transmission in cultured hippocampal neurons. J Biol Chem.

[49] Zhou F, Yang X, Zhao H, Liu Y, Feng Y, An R (2018). Down-regulation of OGT promotes cisplatin resistance by inducing autophagy in ovarian cancer. Theranostics.

[50] Makino Y, Polygalov D, Bolanos F, Benucci A, McHugh TJ (2019). Physiological Signature of Memory Age in the Prefrontal-Hippocampal Circuit. Cell Rep.

[51] Moscovitch M, Cabeza R, Winocur G, Nadel L (2016). Episodic Memory and Beyond: The Hippocampus and Neocortex in Transformation. Annu Rev Psychol.

[52] Squire LR (1992). Memory and the hippocampus: a synthesis from findings with rats, monkeys, and humans. Psychol Rev.

[53] Godsil BP, Kiss JP, Spedding M, Jay TM (2013). The hippocampal-prefrontal pathway: the weak link in psychiatric disorders?. Eur Neuropsychopharmacol.

[54] Barker GR, Banks PJ, Scott H, Ralph GS, Mitrophanous KA, Wong LF (2017). Separate elements of episodic memory subserved by distinct hippocampal-prefrontal connections. Nat Neurosci.

[55] Eichenbaum H (2017). Prefrontal-hippocampal interactions in episodic memory. Nat Rev Neurosci.

[56] Sankowski R, Strohl JJ, Huerta TS, Nasiri E, Mazzarello AN, D'Abramo C (2019). Endogenous retroviruses are associated with hippocampus-based memory impairment. Proc Natl Acad Sci U S A.

[57] Euston DR, Gruber AJ, McNaughton BL (2012). The role of medial prefrontal cortex in memory and decision making. Neuron.

[58] Dulas MR, Duarte A (2016). Age-related changes in overcoming proactive interference in associative memory: The role of PFC-mediated executive control processes at retrieval. Neuroimage.

[59] Binder S, Molle M, Lippert M, Bruder R, Aksamaz S, Ohl F (2019). Monosynaptic Hippocampal-Prefrontal Projections Contribute to Spatial Memory Consolidation in Mice. J Neurosci.

[60] Gupta N, Singh SS, Stopfer M (2016). Oscillatory integration windows in neurons. Nat Commun.

[61] Halasz P, Bodizs R, Parrino L, Terzano M (2014). Two features of sleep slow waves: homeostatic and reactive aspects-from long term to instant sleep homeostasis. Sleep Med.

[62] Cao W, Lin S, Xia QQ, Du YL, Yang Q, Zhang MY (2018). Gamma Oscillation Dysfunction in mPFC Leads to Social Deficits in Neuroligin 3 R451C Knockin Mice. Neuron.

[63] Tamura M, Spellman TJ, Rosen AM, Gogos JA, Gordon JA (2017). Hippocampal-prefrontal theta-gamma coupling during performance of a spatial working memory task. Nat Commun.

[64] Barbay M, Diouf M, Roussel M, Godefroy O, group Gs (2018). Systematic Review and Meta-Analysis of Prevalence in Post-Stroke Neurocognitive Disorders in Hospital-Based Studies. Dement Geriatr Cogn Disord.

[65] Yu (2013). K. H, Cho. S. J, Oh. M. S, Jung. S, Lee. J. H, Shin. J. H, et al. Cognitive impairment evaluated with Vascular Cognitive Impairment Harmonization Standards in a multicenter prospective stroke cohort in Korea. Stroke.

[66] Akinyemi RO, Allan LM, Oakley A, Kalaria RN (2017). Hippocampal Neurodegenerative Pathology in Post-stroke Dementia Compared to Other Dementias and Aging Controls. Front Neurosci.

[67] Kalaria RN, Akinyemi R, Ihara M (2016). Stroke injury, cognitive impairment and vascular dementia. Biochim Biophys Acta.

[68] Pasi M, Poggesi A, Salvadori E, Pantoni L (2012). Post-stroke dementia and cognitive impairment. Front Neurol Neurosci.

[69] Teuschl Y, Matz K, Brainin M (2013). Prevention of post-stroke cognitive decline: a review focusing on lifestyle interventions. Eur J Neurol.

[70] Douiri A, McKevitt C, Emmett ES, Rudd AG, Wolfe CD (2013). Long-term effects of secondary prevention on cognitive function in stroke patients. Circulation.

[71] Akinyemi RO, Allan L, Owolabi MO, Akinyemi JO, Ogbole G, Ajani A (2014). Profile and determinants of vascular cognitive impairment in African stroke survivors: the CogFAST Nigeria Study. J Neurol Sci.

[72] Rapaport D, Fichtman B, Weidberg H, Sprecher E, Horowitz M (2018). NEK3-mediated SNAP29 phosphorylation modulates its membrane association and SNARE fusion dependent processes. Biochem Biophys Res Commun.

[73] Becker-Hapak M, McAllister SS, Dowdy SF (2001). TAT-mediated protein transduction into mammalian cells. Methods.

