# Supplementary material for: Decreased neuronal synaptosome associated protein 29 contributes to poststroke cognitive impairment by disrupting presynaptic maintenance
Source: Theranostics. 2021 Mar 4;11(10):4616–36. doi: 10.7150/thno.54210 (PMC7978312; doi:10.7150/thno.54210)
Supplement: Supplementary file 1 — Supplementary figures. [file thnov11p4616s1.pdf]

Supplementary

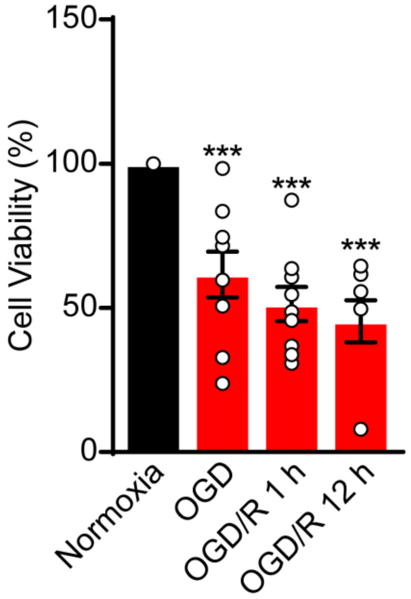

Figure S1. Quantitative analysis of the CCK assay showed the influence of OGD and OGD/R treatment on the survival of cultured cortical neurons (****p* < 0.05, n = 9 per group, one-way ANOVA; Bonferroni post-test). Data are shown as mean and SEM.

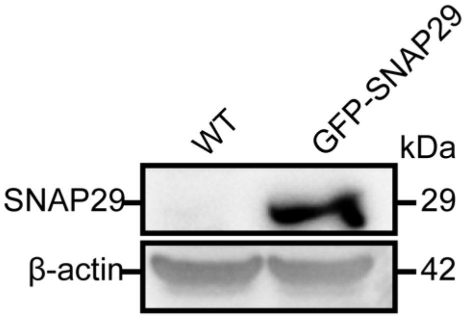

Figure S2. Representative SNAP29 protein levels of cultured cortical neurons after SNAP29 overexpression with lenti-GFP-*Snap29*.

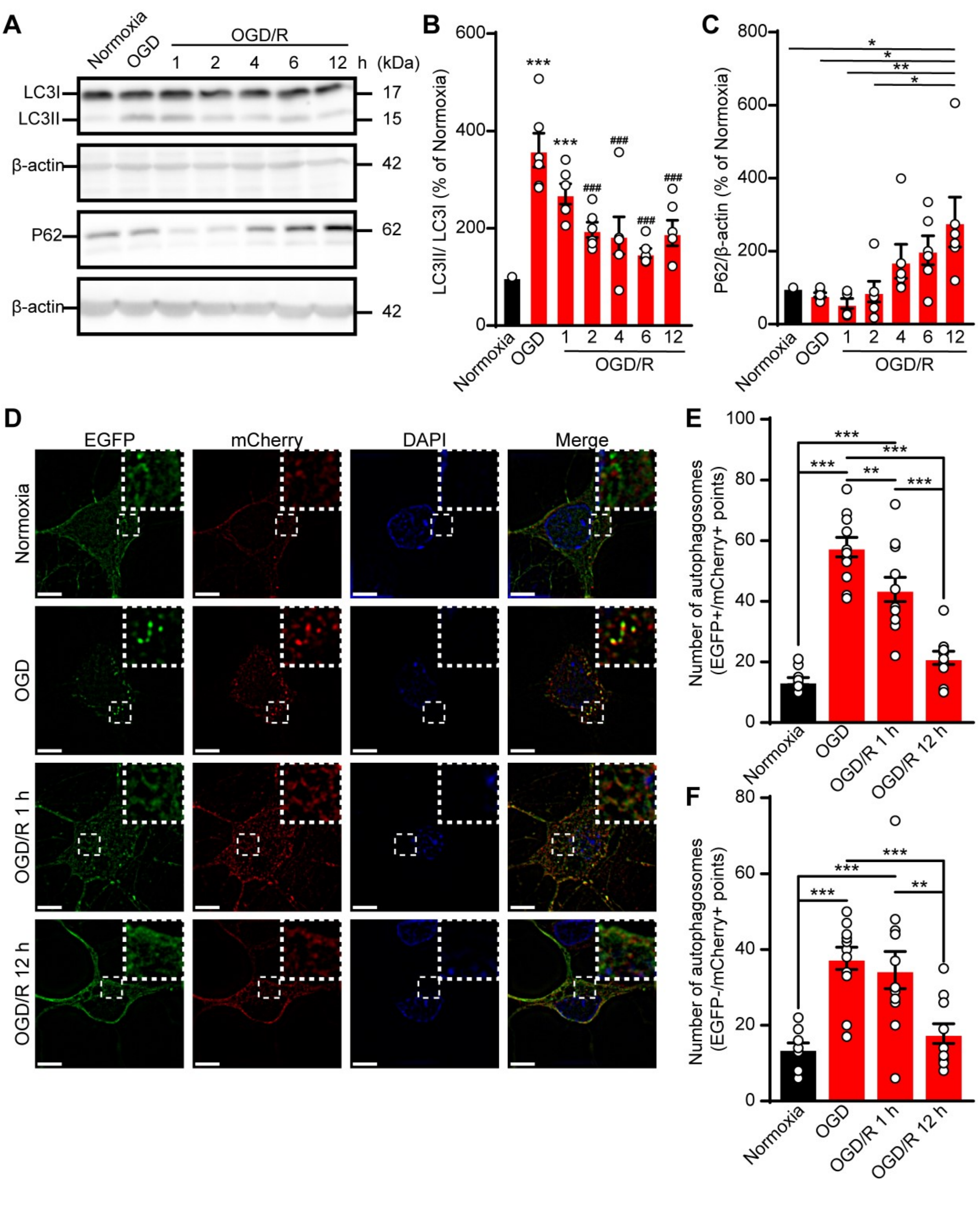

A
Normoxia
OGD
OGD/R
1
2
4
6
12
h
(kDa)
LC3I
LC3II
β-actin
P62
β-actin
17
15
42
62
42
B
LC3II/ LC3I (% of Normoxia)
C
P62/β-actin (% of Normoxia)
D
EGFP
mCherry
DAPI
Merge
OGD/R 1 h
OGD/R 12 h
E
Number of autophagosomes (EGFP+/mCherry+ points)
F
Number of autophagosomes (EGFP-/mCherry+ points)

Figure S3. The OGD or OGD/R-induced decrease of SNAP29 level had no effect on the autophagic flux. **A** LC3 and p62 in neurons was detected after OGD/R exposure with western blot, and quantitative analysis was shown in **B** and **C** (***$p < 0.001$ vs. normoxia group, ##$p < 0.01$ vs. OGD group, n = 6 per group, one-way ANOVA; Bonferroni post-test). **D.** Cortical neurons expressing EGFP-mCherry-LC3 were cultured for 12 days and the numbers of EGFP-mCherry-LC3 and mCherry-LC3 were analyzed. **E.** The numbers of $EGFP^+/mCherry^+$ puncta in neurons following normoxia, OGD, OGD/R 1 h and OGD/R 12 h exposures were presented (**$p < 0.01$, ***$p < 0.001$, n = 10-12 per group, one-way ANOVA; Bonferroni post-test). **F.** The numbers of $EGFP^-/mCherry^+$ puncta in neurons after normoxia, OGD, OGD/R 1 h and OGD/R 12 h exposures were presented (**$p < 0.01$, ***$p < 0.001$, n = 10-12 per group, one-way ANOVA; Bonferroni post-test). Data are shown as mean and SEM.

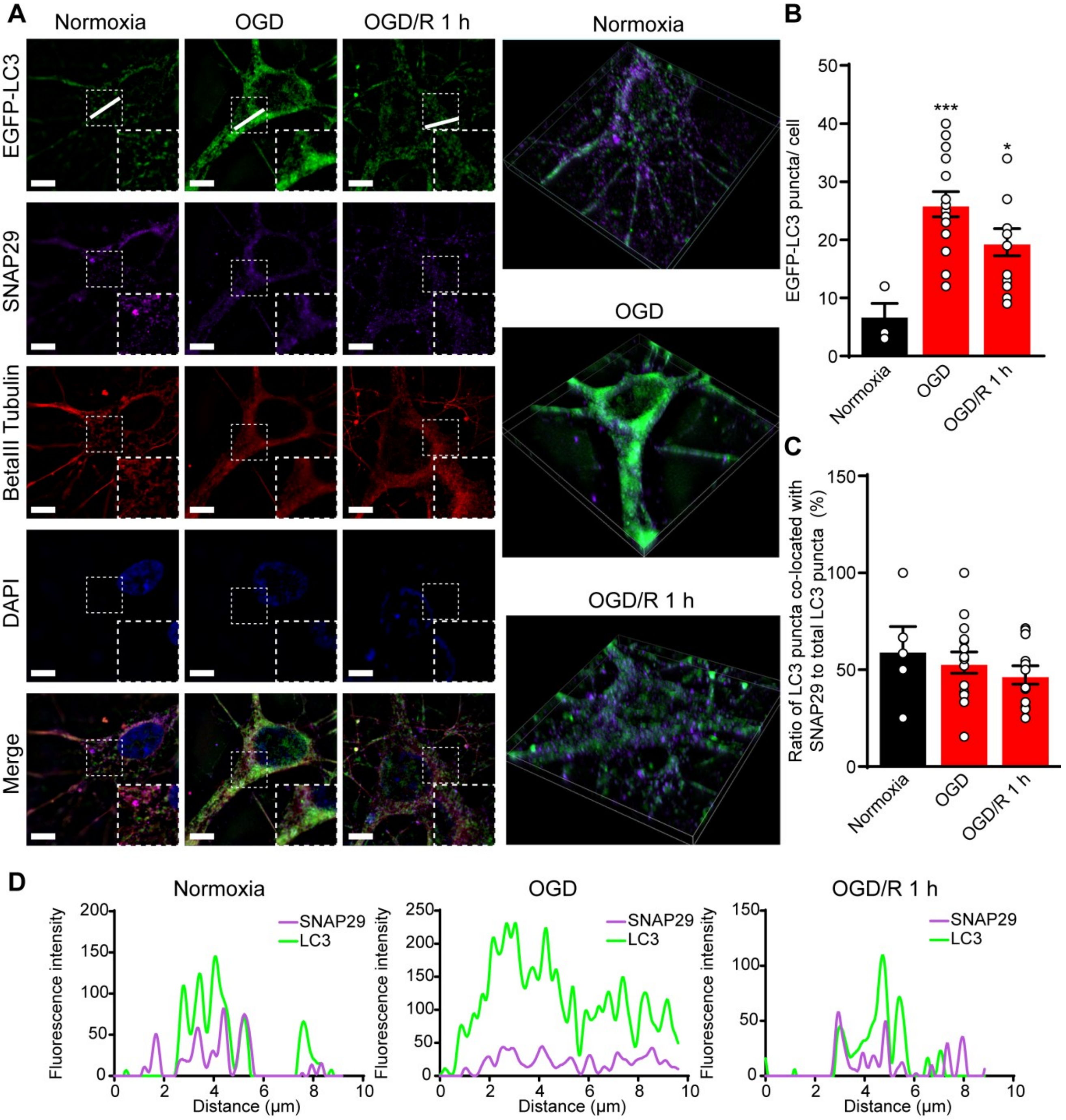

A
Normoxia
OGD
OGD/R 1 h
EGFP-LC3
SNAP29
BetaIII Tubulin
DAPI
Merge
Normoxia
OGD
OGD/R 1 h
B
EGFP-LC3 puncta/ cell
***
*
Normoxia
OGD
OGD/R 1 h
C
Ratio of LC3 puncta co-located with SNAP29 to total LC3 puncta (%)
Normoxia
OGD
OGD/R 1 h
D
Normoxia
OGD
OGD/R 1 h
Fluorescence intensity
SNAP29
LC3
Distance (μm)

Figure S4. The colocalization of SNAP29 and autophagosomes was not influenced by exposure to OGD or OGD/R1 h. **A.** The images of SNAP29 and EGFP-LC3 in neurons in Normoxia, OGD and OGD/R 1 h group were acquired using immunofluorometric assay with SIM microscope (Scale bar: 5 μm). **B.** The number of EGFP-LC3 puncta was detected in Normoxia, OGD and OGD/R 1 h groups (*$p < 0.05$, ***$p < 0.001$, n = 5-12 per group, one-way ANOVA; Bonferroni post-test). **C**. The percentage of EGFP-LC3 puncta colocalizing with SNAP29 was calculated in Normoxia, OGD and OGD/R 1 h groups (one-way ANOVA; Bonferroni post-test). **D.** Line tracings correspond to marked area were used for detecting the colocalization between LC3-positive autophagosomes and SNAP29 (white line in **A**). Data are shown as mean and SEM.

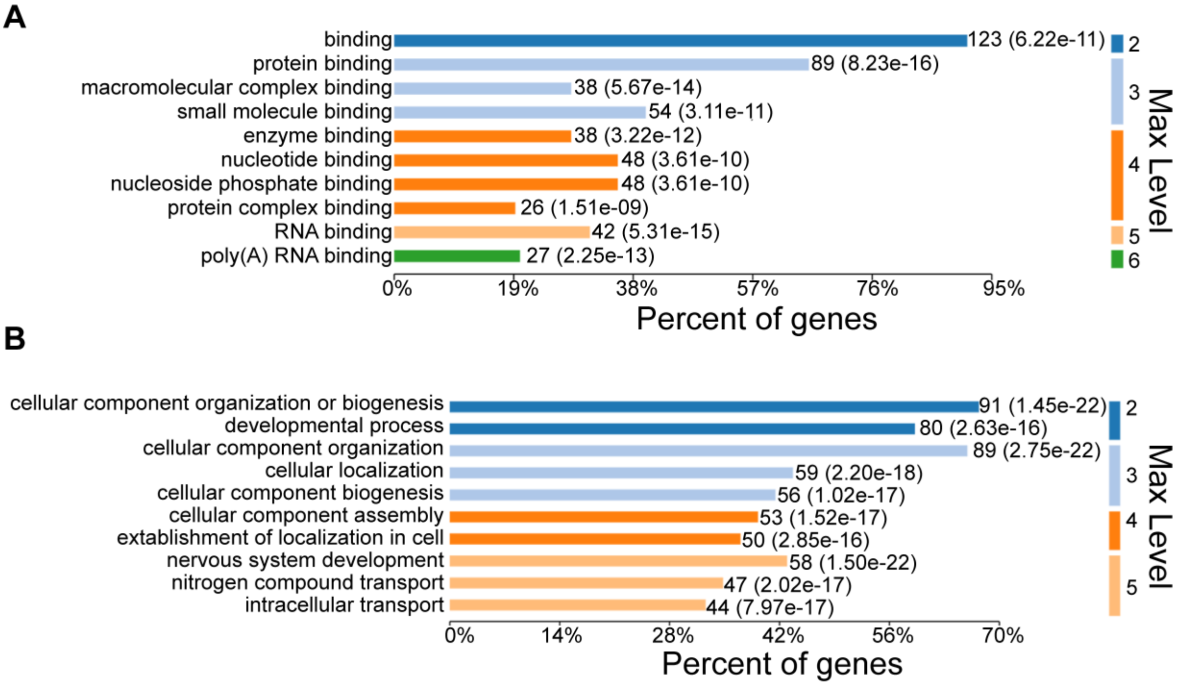

Figure S5. Gene ontology (GO) analysis of differentially expressed SNAP29-interacting proteins. **A.** Differentially expressed SNAP29-interacting proteins were mainly associated with protein-protein binding and small molecule binding. **B.** Differentially expressed SNAP29-interacting proteins were particularly enriched in cellular component organization, cellular localization and developmental process.

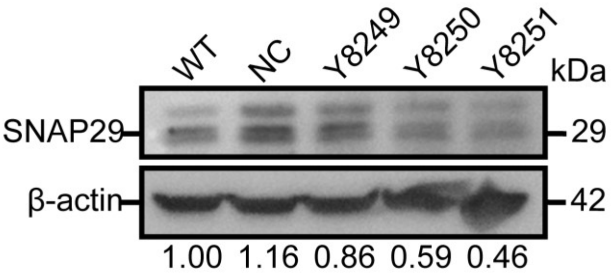

Figure S6 Representative results of SNAP29 knockdown with lenti-shRNA-*Snap29* treatment. Among the three shRNAs, shRNA Y8251 was used in subsequent experiments based on its superior efficiency. Quantitative western blot data shown as the numbers under the blots represent the protein levels relative to WT group.

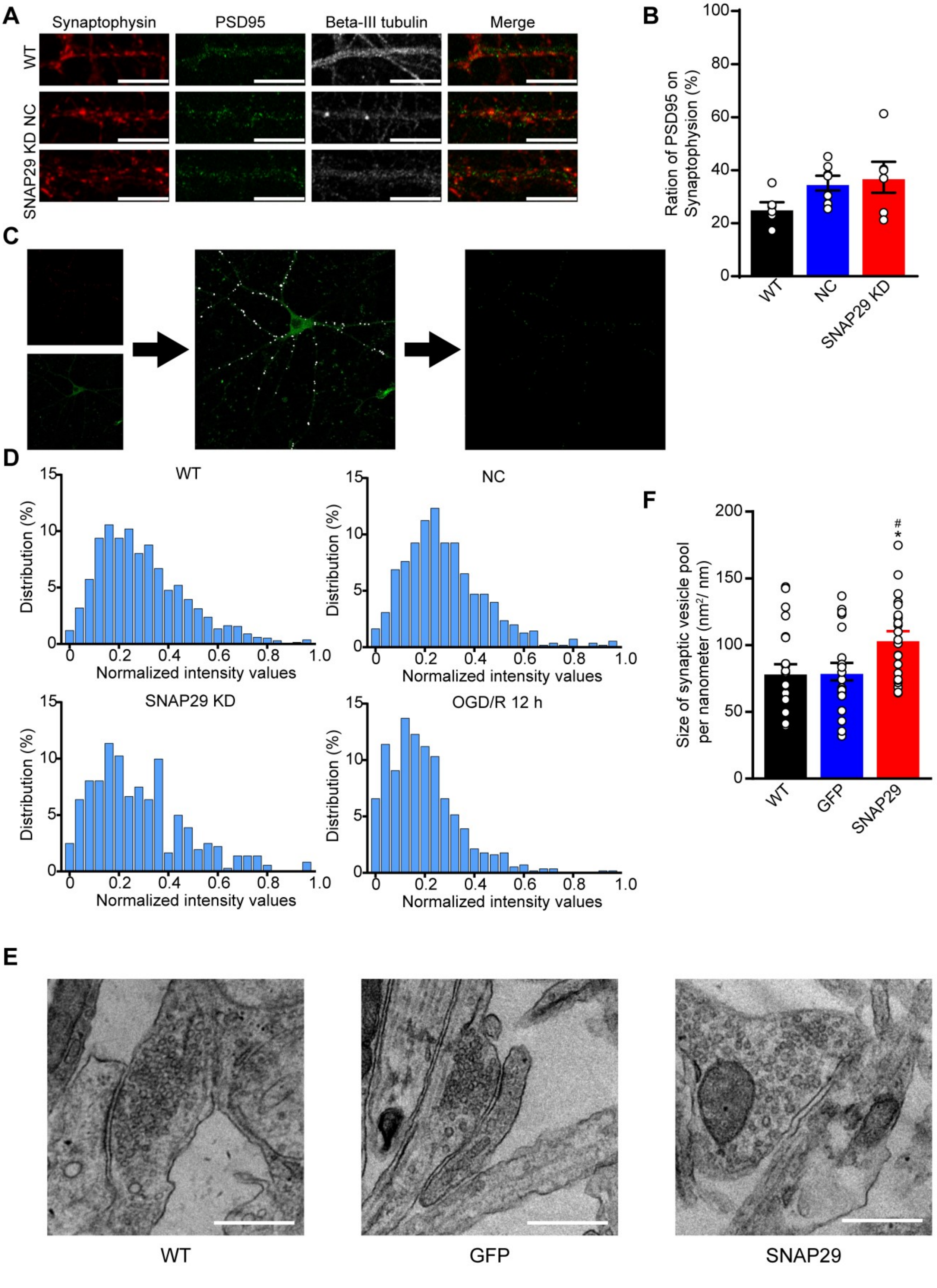
A
Synaptophysin
PSD95
Beta-III tubulin
Merge
WT
SNAP29 KD NC
B
Ration of PSD95 on Synaptophysion (%)
WT
NC
SNAP29 KD
C
D
WT
NC
SNAP29 KD
OGD/R 12 h
Distribution (%)
Normalized intensity values
F
Size of synaptic vesicle pool per nanometer (nm²/ nm)
WT
GFP
SNAP29
E
WT
GFP
SNAP29

Figure S7 SNAP29 KD didn't influence the number of synapses, while SNAP29 distribution at the presynaptic sites were acquired. **A**. The colocalization of PSD95 and synaptophysin was present in neurons with SNAP29 transfection (Scale bar: 10 μm). **B.** The percentages of SNAP29 colocalizing with synaptophysin and PSD95 colocalizing with synaptophysin were expressed in neurons after shRNA transfection ($^{**}p < 0.01$ vs. WT group, $^{\#}p < 0.05$ vs. NC group, n = 6 per group, one-way ANOVA; Bonferroni post-test). **C.** Representative confocal images of processes of cortical neurons stained with SNAP29 and synaptophysin (top left panel). The distribution of synaptophysin (white points) and SNAP29 (green fluorescence) was acquired (top middle panel). Note the distribution of SNAP29 with synaptophysin puncta (top right panel). **D.** The distribution plots of normalized intensity value of synaptophysin compared with SNAP29 were acquired. **E.** The images of synaptic ultrastructure of neurons were acquired with TEM in WT, GFP and SNAP29 overexpressed groups. **F.** The size of synaptic vesicle pool was calculated ($^{*}p < 0.05$ vs. WT group, $^{\#}p < 0.05$ vs. GFP group, n = 22-23 per group, one-way ANOVA; Bonferroni post-test)**.** Data are shown as mean and SEM.

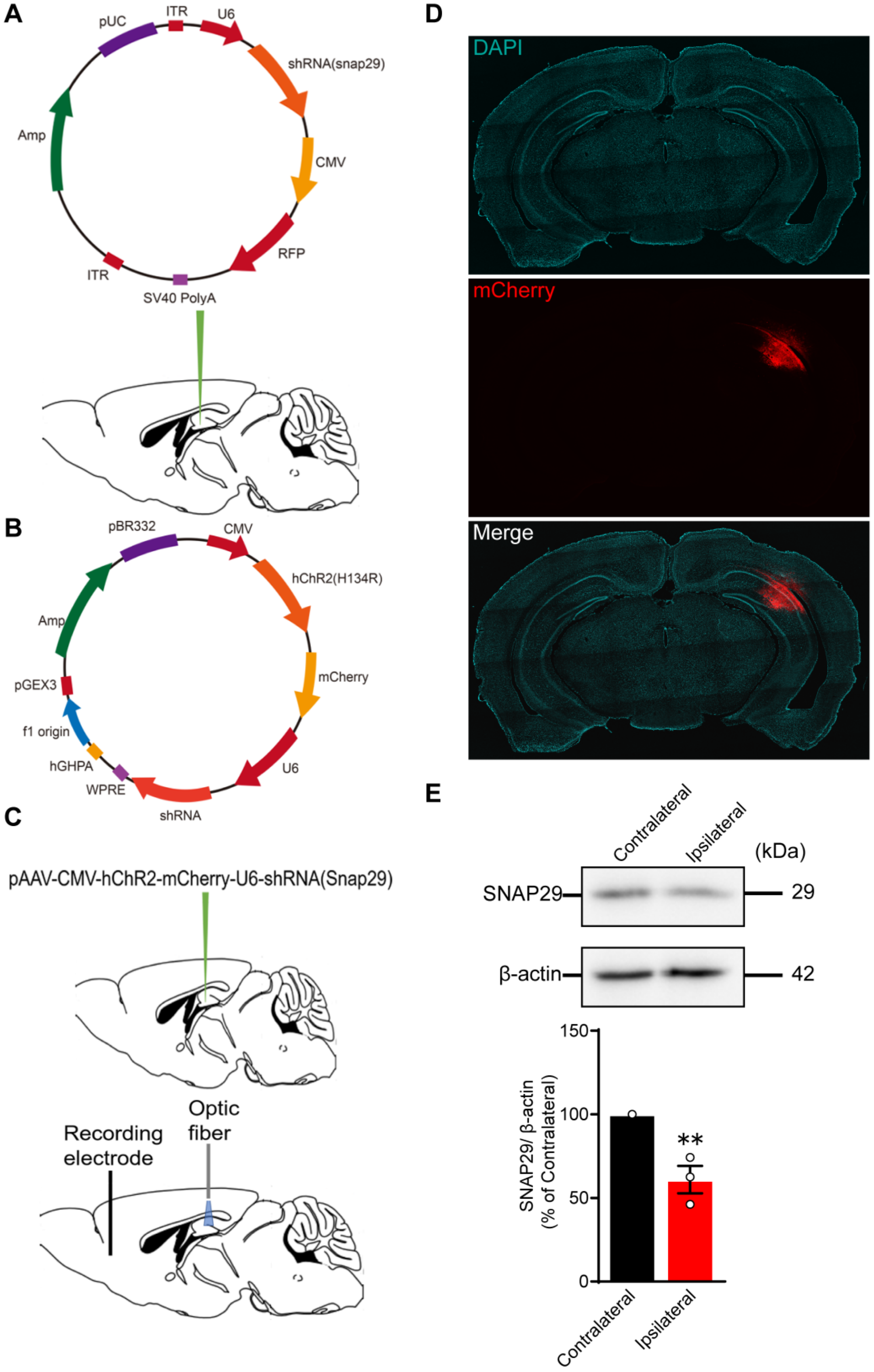
A
pUC
ITR
U6
shRNA(snap29)
Amp
CMV
RFP
ITR
SV40 PolyA
B
pBR332
CMV
hChR2(H134R)
Amp
mCherry
pGEX3
f1 origin
hGHPA
WPRE
U6
shRNA
C
pAAV-CMV-hChR2-mCherry-U6-shRNA(Snap29)
Recording electrode
Optic fiber
D
DAPI
mCherry
Merge
E
Contralateral
Ipsilateral
(kDa)
SNAP29
29
β-actin
42
SNAP29/ β-actin (% of Contralateral)
150
100
50
0
**
Contralateral
Ipsilateral

Figure S8 The AAV vectors carrying *Snap29* shRNA or hChR2(H123R)-mCherry-U6-shRNA were injected into CA1 region of hippocampus in the TEM microscopy and behavioral tests, or optogenetics experiments. **A**. AAVs carrying *Snap29* shRNA were injected (shown in green) into CA1 region of hippocampus of WT mice**. B.** The AAV construct used in optogenetics experiments was pAAV-CMV-hChR2(H123R)-mCherry-U6-shRNA (*Snap29*). **C.** Virus was injected into CA1 region of hippocampus bilaterally. Recording electrode was placed in medial prefrontal cortex and optical fiber was placed in hippocampus. **D.** Representative images of AAV used in optogenetics experiments transduction. **E.** After AAV transduction**,** SNAP29 protein levels in ipsilateral and contralateral cerebral regions of were detected with western blot assay, and quantitative analysis was shown. (**p < 0.01vs contralateral cerebral regions, n = 3 per group, Student's t-test; one-tailed)**.** Data are shown as mean and SEM.
